# Maternal deprivation and adolescent alcohol exposure induce sex-dependent alterations in stress-related behavior and lipid signaling in rats

**DOI:** 10.1186/s13293-026-00937-2

**Published:** 2026-06-07

**Authors:** Laura Sánchez-Marín, Adriana Castro-Zavala, María Flores-López, Ana Gavito, Raquel Reviriego, Eva M. Marco, Francisco J. Pavón-Morón, Fernando Rodríguez de Fonseca, Antonia Serrano

**Affiliations:** 1 IBIMA Plataforma Bionand, Instituto de Investigación Biomédica de Málaga, Málaga, Spain; 2 Unidad de Gestión Clínica de Salud Mental, Hospital Regional Universitario de Málaga, 29010 Málaga, Spain; 3 Neurobiology of Memory and Addiction Laboratory, Departamento de Psicobiología y Metodología de Las Ciencias del Comportamiento, Universidad de Málaga, Málaga, Spain; 4 Facultad de Ciencias Biológicas, Departamento de Genética, Fisiología y Microbiología, Universidad Complutense, Madrid, Spain; 5 Área del Corazón, Hospital Universitario Virgen de La Victoria, Málaga, Spain; 6 CIBER de Enfermedades Cardiovasculares (CIBERCV), Instituto de Salud Carlos III, Madrid, Spain; 7 Unidad de Gestión Clínica de Neurología, Hospital Regional Universitario de Málaga, 29010 Málaga, Spain

**Keywords:** Maternal deprivation, Adolescence, Alcohol, Sexual dimorphism, Lipid mediators

## Abstract

**Background:**

Early-life stress constitutes a major risk factor for the development of neuropsychiatric and substance use disorders, exerting enduring effects on stress responsivity and emotional behavior. Maternal deprivation (MD) is a well-established model of early adversity that induces persistent neurobiological alterations. During adolescence, alcohol exposure presents an additional challenge that may interact with early-life stress, potentially influencing long-term adaptations in a sex-dependent manner. Among the systems involved, lipid signaling pathways, including the endocannabinoid system (ECS) and lysophosphatidic acid (LPA) signaling, have emerged as key regulators of stress adaptation.

**Methods:**

Male and female Wistar rats were subjected to a single 24-h MD episode on postnatal day 9 (PND9). During adolescence (PND31–55), animals received intermittent alcohol exposure (3 g/kg) or saline. Behavioral assessments included the forced swim test and elevated plus maze. Plasma levels of corticosterone, monoacylglycerols, N-acylethanolamines, LPA, and autotaxin were measured. Concurrently, mRNA expression of genes encoding ECS- and LPA-related receptors, as well as enzymes involved in the metabolism of these lipid signaling pathways, was analyzed in the medial prefrontal cortex (mPFC). Data were evaluated using three-way ANOVA with sex, MD, and adolescent alcohol exposure as factors.

**Results:**

MD induced persistent metabolic alterations and revealed sex-specific effects on alcohol pharmacokinetics, with increased blood alcohol concentrations in MD females. Behavioral analyses demonstrated sex-dependent effects, with MD increasing active coping in males and decreasing it in females, while enhancing open-arm exploration independently of adolescent alcohol exposure. Adolescent alcohol exposure increased plasma corticosterone levels. MD and sex significantly altered circulating lipid mediators, showing opposite patterns in males and females. Additionally, MD and adolescent alcohol exposure differentially modulated the expression of ECS- and LPA-related genes in the mPFC, indicating sex-dependent molecular adaptations.

**Conclusions:**

Early-life stress establishes long-lasting, sex-specific alterations in behavioral and lipid-mediated stress responses. Adolescent alcohol exposure acts as a secondary challenge, unmasking or amplifying these adaptations. The integration of peripheral lipid profiles with mPFC molecular changes highlights lipid signaling pathways as key mediators linking early adversity and adolescent experiences with long-term vulnerability to stress-related psychopathology.

**Supplementary Information:**

The online version contains supplementary material available at 10.1186/s13293-026-00937-2.

## Highlights

- Early-life stress induces enduring, sex-specific alterations in behavior, stress responses, and lipid signaling.
- Maternal deprivation selectively modifies stress-coping strategies and circulating lipid mediators in males and females.
- Adolescent alcohol exposure elevates corticosterone levels and unveils or intensifies pre-existing alterations triggered by early-life stress.
- Endocannabinoid and LPA signaling pathways in the mPFC are subject to differential regulation in a sex-dependent manner.
- These findings underscore lipid signaling as a pivotal mechanism connecting early adversity, adolescent experiences, and susceptibility to stress-related disorders.

**Supplementary Information:**

The online version contains supplementary material available at 10.1186/s13293-026-00937-2.

## Abstract (--)

Early life stress and alcohol use during adolescence can have lasting effects on mental health. These experiences may shape how the brain and body respond to stress later in life, and importantly, these effects can differ between males and females. In our study, we explored how early-life stress and alcohol consumption interact to impact behavior and biological processes in male and female rats. We used a well-established animal model where young rats experienced brief separation from their mothers, simulating early adversity. Later, during adolescence, some of these animals were repeatedly exposed to alcohol. We then examined how these experiences affected the way the animals responded to stressful situations, as well as changes in stress-related hormones and specific lipid molecules in the blood that are involved in regulating stress responses. Additionally, we examined changes in the prefrontal cortex, a brain area key to emotions and decision-making. Our findings revealed that early-life stress caused long-term changes that differed between males and females, influencing how they deal with stress and respond to alcohol. We also observed variations in important molecules involved in stress regulation, again showing different patterns between the sexes. Overall, our findings suggest that early experiences can shape long-term responses to stress and alcohol in different ways in males and females. Understanding these differences may help improve future approaches to preventing and treating stress-related and alcohol-related disorders.

## Background

Early-life stress constitutes a major risk factor for the development of neuropsychiatric and substance use disorders later in life, exerting enduring effects on stress responsivity, emotional behavior, and neurobiological systems involved in adaptation to environmental challenges [1–4]. Maternal deprivation (MD) is a well-established animal model of early-life adversity that induces persistent alterations in neuroendocrine, metabolic, and neural systems that are critical for adaptive response to stress. Brief but severe disruption of mother–offspring interactions during early postnatal development has been shown to permanently alter hypothalamic–pituitary–adrenal (HPA) axis function, leading to dysregulated corticosterone secretion, impaired stress feedback regulation, and heightened stress reactivity later in life [5–7]. Brain regions such as the medial prefrontal cortex (mPFC), hippocampus, and amygdala are particularly sensitive to early-life stress, and MD-induced alterations in these areas have been linked to long-term behavioral phenotypes, including changes in anxiety-like behavior, stress-coping strategies, and vulnerability to substance use [8–11]. Moreover, previous studies have reported sex-specific differences in behavioral and neurobiological responses to MD [12–14], underscoring the need to consider sex as a biological variable when investigating the long-term impact of MD.

Adolescence represents a critical developmental period characterized by neurobiological, hormonal, and behavioral maturation, during which the brain remains highly plastic and susceptible to environmental influences. Early-life stress interacts with adolescent brain development, shaping behavioral trajectories and increasing vulnerability to maladaptive outcomes when additional challenges are encountered [15]. In this context, adolescent alcohol exposure poses a significant additional challenge and has been consistently associated with persistent alterations in emotional behavior, stress regulation, and brain function [16, 17]. A growing body of evidence indicates that alcohol exposure during adolescence disrupts synaptic plasticity, stress hormone signaling, and neuroimmune and metabolic pathways, with effects that often persist into adulthood [16, 18–21]. Importantly, both early-life stress and adolescent alcohol consumption exert lasting adverse effects on brain and behavior, which may occur independently or synergistically. Adolescence is also characterized by heightened sensitivity to stress, during which both environmental challenges and substance exposure, including alcohol, can engage stress-regulatory systems, and repeated or sequential stress exposures may further exacerbate neurobiological and behavioral adaptations. Consequently, previous studies suggest that cumulative or sequential stressors during adolescence may interact with early-life adversity to exacerbate long-term behavioral and neurobiological alterations [22, 23]. This framework is consistent with “two-hit” models, which propose that early-life insults can increase vulnerability to subsequent environmental challenges [24], and more broadly with multi-hit concepts of vulnerability and resilience [25]. In this regard, early-life events may prime neurobiological systems, enhancing their sensitivity to later stressors and increasing the risk of long-term behavioral and neurobiological alterations [26]. Moreover, sex differences in brain maturation, hormonal balance, and stress responsiveness during adolescence imply that males and females may differ in their vulnerability and adaptive responses to repeated challenges during this developmental stage [27].

Among the neurobiological systems involved in stress- and alcohol-related neuroadaptations, the endocannabinoid system (ECS) plays a central role in regulating emotional behavior, HPA axis activity, metabolic homeostasis, and neuroimmune function [for review see [28–31]]. This modulatory system comprises cannabinoid receptors, their endogenous lipid ligands, and the enzymes responsible for their synthesis and degradation [32]. Endocannabinoid-related lipid mediators, including monoacylglycerols and N-acylethanolamines derived from common fatty acid precursors, are tightly regulated and exhibit dynamic responses to stress and alcohol exposure [28, 33, 34]. Concurrently, the lysophosphatidic acid (LPA) signaling system, which is metabolically and functionally linked to the ECS [35–38], has emerged as an important modulator of neurodevelopment, synaptic plasticity, and neuroinflammatory processes [39]. Alterations in LPA signaling, such as changes in circulating LPA levels, autotaxin activity, and LPA receptor expression, have been associated with stress-related phenotypes and substance exposure [40–45]. Despite growing evidence implicating both ECS- and LPA-related lipid signaling in stress and alcohol-induced neuroadaptations, their coordinated regulation following early-life stress and adolescent alcohol exposure, particularly in a sex-dependent manner, remains poorly understood.

This study seeks to examine how early-life stress and adolescent alcohol exposure interact to influence behavioral, endocrine, and lipid signaling alterations in male and female rats. To this end, plasma levels of corticosterone, endocannabinoid-related lipid mediators, and components of the LPA signaling system were analyzed to assess systemic adaptations to stress and metabolic regulation. Concurrently, gene expression of cannabinoid and LPA receptors, as well as key lipid-metabolizing enzymes, was investigated in the mPFC. This brain region plays a central role in the integration of stress-related signals, executive function, and emotional regulation, and exerts top-down control over subcortical structures such as the amygdala and hypothalamus, thereby modulating both emotional reactivity and HPA axis activity. Moreover, the mPFC is highly sensitive to both early-life stress and adolescent alcohol exposure, which can induce long-lasting alterations in synaptic plasticity, stress responsiveness, and behavioral regulation, making it a key region for investigating stress-related neuroadaptations. By integrating peripheral and central measures, this research aims to elucidate sex-specific molecular and behavioral adaptations through which early-life stress and adolescent alcohol exposure modulate stress-related phenotypes.

## Methods

### Animals

Experimental animals were obtained from timed matings of adult Wistar rats purchased from Charles River Laboratories (Wilmington, MA, USA). The rats were maintained under standard housing conditions with controlled temperature (21 ± 1 °C) and humidity (55% ± 10%), on a 12-h light/dark cycle (lights off at 8:00 h), with unrestricted access to food and water. Following a seven-day acclimation period, breeding was conducted by housing one male with two females for five consecutive days, ensuring at least one complete estrous cycle. Subsequently, males were removed, and pregnant females were housed individually and monitored daily. The day of delivery was designated as postnatal day 0 (PND0). A total of 65 offspring were included in the study (36 males and 29 females).

### Experimental design and procedures

**Fig. 1:**
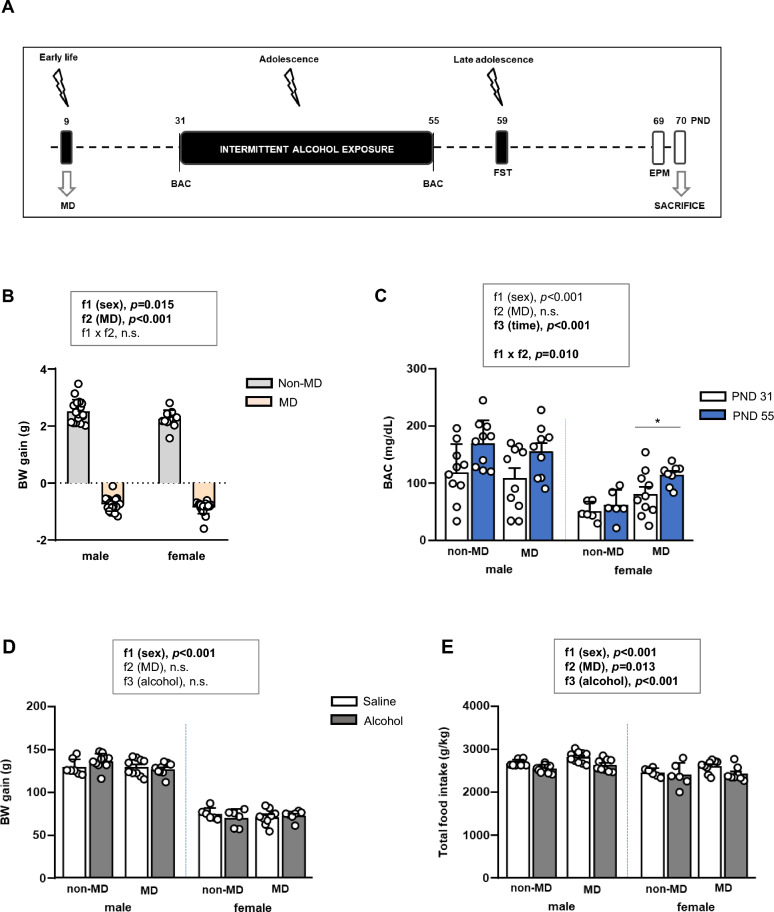
Effects of maternal deprivation, adolescent alcohol exposure, and sex on body weight, food intake, and blood alcohol concentration. (**A**) Experimental design illustrating maternal deprivation (MD) at postnatal day (PND) 9 and intermittent alcohol exposure during adolescence (PND31–55). Animals were assigned to control or MD conditions followed by alcohol or saline treatment. (**B**) Total body weight gain following MD. (**C**) Blood alcohol concentration (BAC) measured after the first and last alcohol administration. (**D**) Body weight gain and (**E**) total food intake during adolescent alcohol exposure. Data are expressed as mean ± SEM. Statistical analyses were performed using two-way ANOVA (panel B) or three-way ANOVA (panels C-E). *p*-values in bold denote significant main effects of factors or significant interaction. Symbols indicate significant effects: (*) denotes *p* < 0.05, comparing MD rats to non-MD rats in males or females

The experimental design is shown in Fig. 1A. Animals were obtained from 14 dams (paired with 7 males for breeding), generating a total of 69 offspring, of which 65 were included in the final analyses. Offspring from different litters were distributed across experimental groups to minimize potential litter effects. Male and female rats were assigned to either control or MD conditions, followed by intermittent exposure to alcohol or saline. This resulted in a total of eight experimental groups: non-MD saline males (*n* = 7 from 4 dams), non-MD saline females (*n* = 6 from 3 dams), non-MD alcohol males (*n* = 10 from 4 dams), non-MD alcohol females (*n* = 6 from 4 dams), MD saline males (*n* = 10 from 5 dams), MD saline females (*n* = 9 from 5 dams), MD alcohol males (*n* = 9 from 4 dams), and MD alcohol females (*n* = 8 from 4 dams).

#### Maternal deprivation (MD)

To model early-life stress, half of the litters (19 males and 17 females) were separated from their dams for 24 h on PND9, as previously described [46]. Mothers were removed at 09:00 h, whereas pups stayed in their home cage with transferred nest bedding. During separation, mother and pups were housed in adjacent locations within the vivarium. No food or heating was provided to the pups during this deprivation period. Mothers were returned to their home cages with their respective litters 24 h later. Control animals (17 males and 12 females) underwent the same handling except for the MD episode. Body weights of all pups were recorded just before and after the MD episode (PND9 and PND10), as well as one week later.

#### Intermittent alcohol exposure during adolescence

During adolescence, rats were subjected to intermittent ethanol administration via intragastric gavage between PND31 and PND55, in accordance with previously established protocols [18–20, 47]. In brief, animals designated for the alcohol condition (MD: 9 males and 8 females; non-MD: 10 males and 6 females) received ethanol at a dose of 3 g/kg (25% v/v in saline; 15 mL/kg) for four consecutive days, followed by a three-day period of alcohol deprivation. This cycle was repeated over a four-week period. Control animals (MD: 10 males and 9 females; non-MD: 7 males and 6 females) received equivalent volumes of saline according to the same schedule. Throughout the exposure period, body weight and food intake were monitored weekly. Food intake was measured at the cage level and normalized per animal, as rats were housed in pairs. Intake values were further normalized to body weight and expressed as g/kg.

### Blood alcohol concentration (BAC)

To assess alcohol exposure, tail blood samples were collected one hour after the first and last administrations of ethanol or saline. Samples were centrifuged at 2000 × g for 15 min to isolate plasma. BAC was subsequently determined by an alcohol oxidase-based method using an AM1 Alcohol Analyser (Analox Instruments, London, UK).

### Behavioral procedures

All tests were conducted at ambient temperature between 10:00 and 15:00 h during the dark phase of the light–dark cycle. To minimize behavioral disturbances, the animals were transported to an adjacent anteroom at least two hours prior to testing. Each rat was placed in the testing apparatus, and their behavior was recorded via digital video. Behavioral assessments were carried out by trained observers blinded to the experimental conditions. A 10-day interval was included between the forced swim test (FST) and the elevated plus maze (EPM) to reduce the impact of acute stress induced by the FST on subsequent behavioral assessments.

#### Forced swim test (FST)

Four days after the last alcohol exposure, rats were subjected to the FST to assess stress-coping behavior. Rats were individually placed in an inescapable transparent cylindrical tank (height × diameter: 50 × 20 cm) filled with water (25 ± 1 °C), and recorded in the tank for 6 min. At the end of the testing period, the animals were dried with paper towels and a heat lamp to prevent hypothermia, then returned to their home cages. Water in the tank was changed between each animal. Immobility and escape behaviors (swimming + climbing) times were measured during the last 4 min of the session.

#### Elevated plus-maze (EPM)

Two weeks after the last exposure to alcohol or saline, anxiety-like behaviors were assessed using the EPM as previously described [19]. The apparatus consisted of two open arms (45 × 10 cm) and two closed arms of the same size with 50 cm-high walls, positioned opposite each other and connected by a central neutral area (10 × 10 cm). Rats were placed in the central area and allowed to explore freely for 5 min. The EPM was cleaned with a solution and dried with paper towels between each test. The following parameters were analyzed: the percentage of time spent in the open arms (time in open arms divided by total time in open and closed arms, multiplied by 100) (open time ratio, OTR); distance traveled in the open arms (cm); number of entries into the open arms; time spent in the closed arms (s); total distance traveled (cm); and time spent in the center (s). All parameters were quantified using EthoVision XT 17 software (Noldus, Wageningen, The Netherlands).

### Sample collection: blood processing and brain dissection

Two weeks after completing adolescent alcohol exposure (PND70), rats were deeply anesthetized with sodium pentobarbital (50 mg/kg, i.p.), and blood and brain tissue were collected. Blood samples were centrifuged at 2000 × g for 15 min, and plasma was aliquoted and stored for later analyses. Brains were rapidly removed, frozen, and sliced into 2-mm coronal sections using a rat brain matrix. The medial prefrontal cortex (mPFC), including the anterior cingulate, prelimbic, and infralimbic cortices, was dissected according to the Paxinos and Watson atlas [48] between + 3.70 and + 2.20 mm anterior to bregma (corresponding to interaural coordinates 12.70–11.20 mm). Tissue samples were kept at -80 ºC until molecular and biochemical analyses were conducted.

### Determinations in plasma

#### Corticosterone, LPA, and autotaxin

Commercially available enzyme-linked immunoassay (ELISA) kits were used to measure plasma levels of corticosterone (ab323743, Abcam Ltd., Cambridge, UK), LPA (MBS2612504, MyBioSource, Inc., San Diego, CA, USA), and autotaxin (SEC323Ra, Cloud‑Clone Corp., Katy, TX, USA), following the manufacturer’s instructions. Data were expressed as nanograms of protein per milliliter of plasma (ng/mL).

#### Monoacylglycerols and N-acylethanolamines

The quantification of fatty acid-derived molecules in plasma samples was performed using liquid chromatography–tandem mass spectrometry (LC–MS/MS), following a previously validated method [20, 49]. The following monoacylglycerols and N-acylethanolamines were measured: 2-arachidonoylglycerol (2-AG), 2-oleoylglycerol (2-OG), 2-linoleoylglycerol (2-LG), oleoylethanolamide (OEA), linoleoylethanolamide (LEA), and arachidonoylethanolamide (AEA). Briefly, plasma samples (0.5 mL) were transferred to glass tubes and spiked with 25 μL of an acetonitrile mixture containing deuterated internal standards. Next, samples were diluted with 0.5 mL of 0.1 M ammonium acetate buffer (pH 4.0) and extracted with 6 mL of tert-butyl methyl ether. The samples were centrifuged at 2800 × g for 5 min, and the organic extracts were reconstituted in 100 μL of a water/acetonitrile mixture (10:90, v/v) with 0.1% formic acid, and subsequently transferred to HPLC vials. A volume of 20 μL was injected into a liquid chromatography-tandem mass spectrometry system (LC–MS/MS). An Agilent 6410 triple quadrupole (Agilent Technologies, Wilmington, DE, USA) equipped with a 1200 series binary pump, a column oven, and a cooled auto-sampler (4 ºC) was used. The chromatographic separation was conducted using an ACQUITY UPLC C18-CSH column (3.1 × 100 mm, 1.8 μm particle size) (Waters, Yvelines Cedex, France) maintained at 40 ºC with a mobile phase flow rate of 0.4 mL/min. The mobile phase comprised the following constituents: A) 0.1% (v/v) formic acid in water and B) 0.1% (v/v) formic acid in acetonitrile. The relative quantification of the fatty acid derivatives was performed by isotope dilution. Standards were obtained from Cayman Chemical (Ann Arbor, MI, USA) and from Toronto Research Chemicals (North York, ON, Canada). Solvents were purchased from Merck (Darmstadt, Germany).

### RNA isolation and relative quantification for gene expression

Real-time quantitative PCR (RT-qPCR) was used to measure the relative mRNA expression levels of genes involved in endocannabinoid and LPA signaling, as previously described [19, 21, 42].

Total RNA was extracted from brain tissue using TRIzol™ Reagent (Gibco BRL Life Technologies, Baltimore, MD, USA). RNA concentration and purity were measured by spectrophotometry, with a 260/280 absorbance ratio between 1.8 and 2.0. Reverse transcription was performed using iScript™ Reverse Transcription Supermix (Bio Rad Laboratories, Hercules, CA, USA) with random hexamer primers. RT-qPCR reactions were carried out on a CFX Duet Real-Time PCR System (Bio Rad Laboratories, Hercules, CA, USA) using pre-designed TaqMan Gene Expression Assays labeled with FAM dye (Applied Biosystems, Foster City, CA, USA). Gene expression analyses included receptors involved in endocannabinoid and LPA signaling (*Cnr1, Cnr2, Ppara,* and *Lpar1*), and enzymes involved in the synthesis and degradation of these lipid mediators (*Napepld, Dagla, Daglb, Enpp2, Faah*, and *Mgll*).

Primer sequences and assay IDs were obtained from the Applied Biosystems rat mRNA references database (http://bioinfo.appliedbiosystems.com/genome-database/gene-expression.html) (**Table S1**).

Individual samples were analyzed in duplicate, and the mean fluorescence values were calculated. Absolute values were normalized to the housekeeping gene β-actin (*Actb*), which remained stable across groups in the mPFC. Relative quantification was calculated using the 2∧-ΔΔCt method, with expression levels normalized to the non-MD saline male group.

### Statistical analysis

Data are presented as the mean ± standard error of the mean (SEM). The normality of the data distribution was examined using the Kolmogorov–Smirnov test. Differences within and between groups were assessed with a three-way analysis of variance (ANOVA) with ‘sex’ (f1, levels: ‘male’ and ‘female’), ‘MD’ (f2, levels: ‘non-MD’ and ‘MD’), and ‘adolescent alcohol exposure’ (f3, levels: ‘saline’ and ‘alcohol’) as fixed factors. When a significant three-way interaction was detected, follow-up two-way ANOVAs were performed separately within each sex, using ‘MD’ and ‘adolescent alcohol exposure’ as factors. Tukey’s post hoc test was applied when appropriate.

Statistical significance was set at *p* < 0.05. Analyses were conducted using GraphPad Prism version 5.04 software (GraphPad Software, San Diego, CA, USA). Complete ANOVA statistics, including non-significant main effects and interactions, together with effect size estimates (partial eta squared, ηp^2^), are provided in Supplementary Tables S2-S6.

## Results

### Effects of MD, adolescent alcohol exposure, and sex on body weight, food intake, and BAC

#### Body weight after the MD

Body weight changes were first assessed immediately after the MD episode and again one week later. A two-way ANOVA including sex (f1) and MD (f2) as factors revealed a significant main effect of sex [*F* (1,64) = 6.25, *p* = 0.015​], with females showing lower body weight gain than males. Additionally, a significant main effect of MD was observed [*F* (1,64) = 1919, *p* < 0.001], indicating substantial body weight loss in MD rats compared to non-MD rats (Fig. 1B).

One week after the MD episode, MD animals continued to show lower body weight gain compared to non-MD animals. Body weight gain in the different groups was as follows: 15.00 ± 0.38 g in non-MD males, 11.95 ± 0.38 g in MD males, 14.12 ± 0.47 g in non-MD females, and 11.42 ± 0.35 g in MD females.

#### BAC during adolescent alcohol exposure

BAC was then measured in plasma samples collected after the initial and final alcohol administration in male and female rats, and repeated-measures three-way ANOVA with sex (f1), MD (f2), and exposure time (f3) as factors was conducted. The analysis revealed a significant main effect of time [*F* (1,61) = 12.32, *p* < 0.001], indicating higher BAC levels at the end of the adolescent exposure period than after the first administration in both sexes. In addition, a significant sex × MD interaction [*F* (1,61) = 7.06, *p* = 0.010] was detected, showing that BAC was increased in MD females compared to non-MD females, whereas no comparable difference was observed in males (Fig. 1C).

#### Body weight and food intake during adolescent alcohol exposure

Body weight gain and food intake during the adolescent alcohol exposure period were further analyzed by three-way ANOVA with sex (f1), MD (f2), and adolescent alcohol exposure (f3) as factors. For body weight gain, only a significant main effect of sex was observed [*F* (1,57) = 723.3, *p* < 0.001], with males showing greater weight gain than females (Fig. 1D). For food intake, significant main effects of sex [*F* (1,57) = 27.28, *p* < 0.001], MD [*F* (1,57) = 6.61, *p* = 0.013], and adolescent alcohol exposure [*F* (1,57) = 13.79, *p* < 0.001] were found, indicating higher intake in males than in females, higher intake in MD rats than in non-MD rats, and lower intake in alcohol-treated rats than in saline-treated controls (Fig. 1E). Weekly food intake measurements throughout the alcohol exposure period are provided in Supplementary Figure S1.

Complete ANOVA statistics, including non-significant main effects and interactions, together with effect size estimates (ηp^2^), are provided in Supplementary Table S2.

### Effects of MD, adolescent alcohol exposure, and sex on behavior and locomotor activity

We evaluated how MD and adolescent alcohol exposure affect stress-coping behavior, anxiety-like behavior, and locomotor activity in male and female rats. Sex (f1), MD (f2), and adolescent alcohol exposure (f3) were included as factors in the statistical analyses. Complete ANOVA statistics, including non-significant main effects and interactions, together with effect size estimates (ηp^2^), are provided in Supplementary Table S3.

#### Stress-coping behavior

**Fig. 2:**
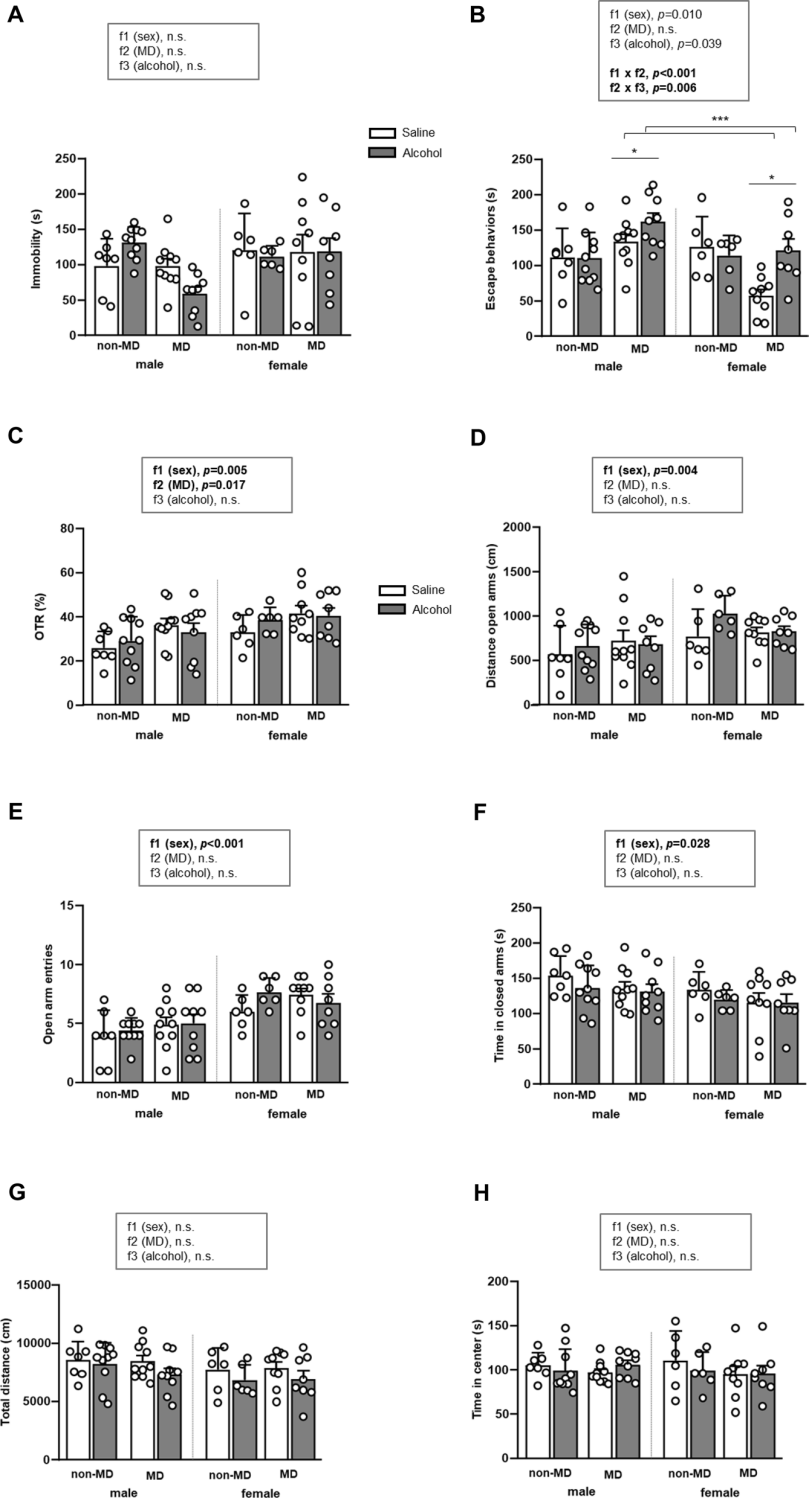
Effects of maternal deprivation, adolescent alcohol exposure, and sex on behavior. Stress-coping behavior assessed in the FST, expressed as (**A**) immobility time and (**B**) escape behavior (swimming + climbing time). Anxiety-like behavior and locomotor activity evaluated in the EPM, expressed as (**C**) the percentage of time spent in the open arms (open time ratio, OTR), (**D**) distance traveled in the open arms, (**E**) number of entries into the open arms, (**F**) time spent in the closed arms, (**G**) total distance traveled, and (**H**) time spent in the center zone. Data are expressed as mean ± SEM. Statistical analyses were performed using three-way ANOVA. *p*-values in bold denote significant main effects of factors or significant interaction. Symbols indicate significant effects: (*) denotes *p* < 0.05, comparing MD rats to non-MD rats in males or females; (***) denotes *p* < 0.05, comparing alcohol-treated rats to saline-treated rats in the MD or non-MD groups

In the FST, immobility time and total climbing plus swimming time (escape behavior) were analyzed. No significant effects were observed for immobility time (Fig. 2A). However, the three-way ANOVA showed significant main effects of sex and adolescent alcohol exposure, along with significant two-way interactions for escape behavior (Fig. 2B). A significant sex × MD interaction was found [*F* (1,57) = 13.53, *p* < 0.001], indicating higher escape behavior in MD males compared to non-MD males; whereas MD females exhibited decreased escape behavior compared to non-MD females. Additionally, a significant MD × alcohol interaction was detected [*F* (1,57) = 8.09, *p* = 0.006], suggesting that alcohol-treated rats displayed higher escape behavior than saline-treated rats within the MD group, but not in the non-MD group.

#### Anxiety-like behavior and locomotor activity

Anxiety-like behavior and locomotor activity were assessed in the EPM. As shown in Fig. 2C, the analysis of OTR revealed significant main effects of sex [*F* (1,57) = 8.74, *p* = 0.005] and MD [*F* (1,57) = 6.11, *p* = 0.017], indicating greater open-arm exploration in females compared to males, and in MD rats compared to non-MD rats. Additionally, a significant main effect of sex was found for the distance traveled in the open arms [*F* (1,57) = 8.79, *p* = 0.004] (Fig. 2D), the number of entries into the open arms [*F* (1,57) = 27.43, *p* < 0.001] (Fig. 2E), and time spent in the closed arms [*F* (1,57) = 5.09, *p* = 0.028] (Fig. 2F), with females showing greater distance traveled and a higher number of entries into the open arms, together with reduced time spent in the closed arms, compared to males. Regarding locomotor activity, total distance traveled did not differ significantly between groups (Fig. 2G). Finally, no significant differences were observed in time spent in the center zone of the maze (Fig. 2H).

### Effects of MD, adolescent alcohol exposure, and sex on circulating stress hormones and lipid mediators

**Fig. 3:**
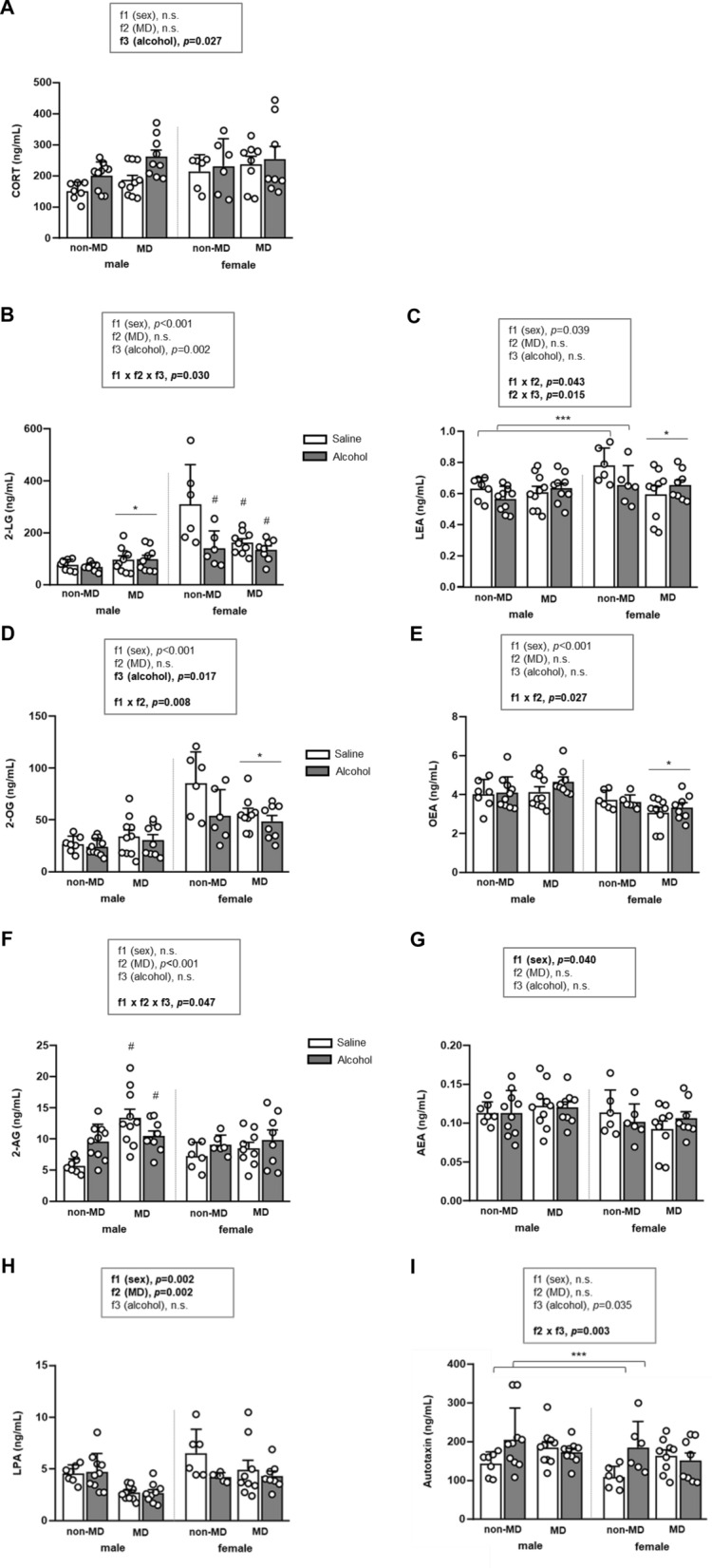
Effects of maternal deprivation, adolescent alcohol exposure, and sex on corticosterone, circulating lipid mediators and LPA signaling. Plasma levels of (**A**) corticosterone; (**B**) 2-linoleoylglycerol (2-LG); (**C**) linoleoylethanolamide (LEA); (**D**) 2-oleoylglycerol (2-OG); (**E**) oleoylethanolamide (OEA); (**F**) 2-arachidonoylglycerol (2-AG); (**G**) arachidonoylethanolamide (AEA); (**H**) lysophosphatidic acid (LPA); and (**I**) autotaxin. Data are expressed as mean ± SEM. Statistical analyses were performed using three-way ANOVA. Where appropriate, significant interactions were further analyzed by two-way ANOVA within each sex. *p*-values in bold denote significant main effects of factors or significant interaction. Symbols indicate significant effects: (*) denotes *p* < 0.05, comparing MD rats to non-MD rats in males or females; (***) denotes *p* < 0.05, comparing alcohol-treated rats to saline-treated rats in the MD or non-MD groups; (#) denotes *p* < 0.05, comparing rats to non-MD saline rats in males or females

The effects of MD and adolescent alcohol exposure on plasma levels of corticosterone, monoacylglycerols, N-acylethanolamines, LPA, and autotaxin were assessed in males and females. Sex (f1), MD (f2), and adolescent alcohol exposure (f3) were included as factors in the statistical analyses (Fig. 3). Complete ANOVA statistics, including non-significant main effects and interactions, together with effect size estimates (ηp^2^), are provided in Supplementary Table S4.

#### Corticosterone levels

As shown in Fig. 3A, the analysis revealed a significant main effect of adolescent alcohol exposure on plasma corticosterone levels [*F* (1,56) = 5.15, *p* = 0.027], with alcohol-treated rats showing higher corticosterone levels than saline-treated rats.

#### Monoacylglycerol and N-acylethanolamine levels

Plasma levels of monoacylglycerols and their corresponding N-acylethanolamines derived from the same fatty acid were measured to assess coordinated regulation of endocannabinoid-related lipid mediators.

For lipids derived from linoleic acid, analysis of 2-LG revealed main effects of sex and adolescent alcohol exposure, along with a significant three-way interaction involving sex, MD, and adolescent alcohol exposure [*F* (1,57) = 4.93, *p* = 0.030] (Fig. 3B). To better understand the MD × alcohol interactions, we analyzed the data separately by sex using two-way ANOVAs. In males, there was a significant main effect of MD [*F* (1,32) = 4.19, *p* = 0.049], with MD rats showing higher 2-LG levels than non-MD rats. In contrast, a significant MD × alcohol interaction was observed in females [*F* (1,25) = 5.46, *p* = 0.028]. Post hoc comparisons showed lower 2-LG levels in all experimental groups compared to non-MD saline females. Regarding plasma levels of the corresponding N-acylethanolamine, LEA, the three-way ANOVA revealed significant main effects of sex and significant two-way interactions (Fig. 3C). A significant sex × MD interaction was detected [*F* (1,57) = 4.30, *p* = 0.043], with MD females showing lower LEA levels compared to non-MD females, whereas no significant differences were observed in male rats. In addition, a significant MD × alcohol interaction was found [*F* (1,57) = 6.26, *p* = 0.015], indicating that alcohol-treated rats showed lower LEA levels than saline-treated rats within the non-MD group, but not within the MD group.

Regarding mediators derived from oleic acid, plasma levels of 2-OG showed a significant main effect of adolescent alcohol exposure [*F* (1,57) = 6.11, *p* = 0.017], with alcohol-treated rats showing lower 2-OG levels than saline-treated rats. Furthermore, a significant sex × MD interaction was observed [*F* (1,57) = 7.58, *p* = 0.008]. Specifically, MD females showed lower 2-OG levels compared to non-MD females, whereas no significant differences were observed in males (Fig. 3D). The analysis of OEA revealed a significant main effect of sex and a significant sex × MD interaction [*F* (1,57) = 5.13, *p* = 0.027] (Fig. 3E), indicating lower OEA levels in MD females than in non-MD females, with no significant difference in males.

For lipids derived from arachidonic acid, analysis of 2-AG revealed a significant main effect of MD and a significant three-way interaction with sex, MD, and adolescent alcohol exposure [*F* (1,57) = 4.14, *p* = 0.047] (Fig. 3F). To further explore this interaction, we analyzed the data separately by sex using two-way ANOVAs. In males, there was a significant MD × alcohol interaction [*F* (1,32) = 10.58, *p* = 0.003], and post hoc comparisons showed higher 2-AG levels in MD males compared to non-MD saline males. In contrast, no significant main effects or interactions were detected in females. As shown in Fig. 3G, the analysis of AEA levels revealed significant main effects of sex [*F* (1,57) = 4.43, *p* = 0.040], indicating lower AEA levels in females than in males.

#### LPA and autotaxin levels

Given the close metabolic and functional relationship between endocannabinoid-related lipids and the LPA signaling pathway, plasma levels of LPA and autotaxin were also examined to assess whether MD and adolescent alcohol exposure differentially affect this lipid signaling system based on sex.

As shown in Fig. 3H, the analysis of LPA levels revealed a significant main effect of sex [*F* (1,57) = 10.96, *p* = 0.002], with females having higher LPA levels compared to males. Additionally, a significant main effect of MD was observed [*F* (1,57) = 11.15, *p* = 0.017], with lower LPA levels in MD rats than in non-MD rats. Regarding plasma autotaxin levels, the three-way ANOVA revealed a significant main effect of adolescent alcohol exposure and a significant MD × alcohol interaction [*F* (1,57) = 9.57, *p* = 0.003] (Fig. 3I). Specifically, alcohol-treated rats had higher autotaxin levels than saline-treated rats within the non-MD group, but not within the MD group.

### Effects of MD, adolescent alcohol exposure, and sex on the mRNA expression of genes encoding receptors of lipid-derived mediators in the mPFC

**Fig. 4:**
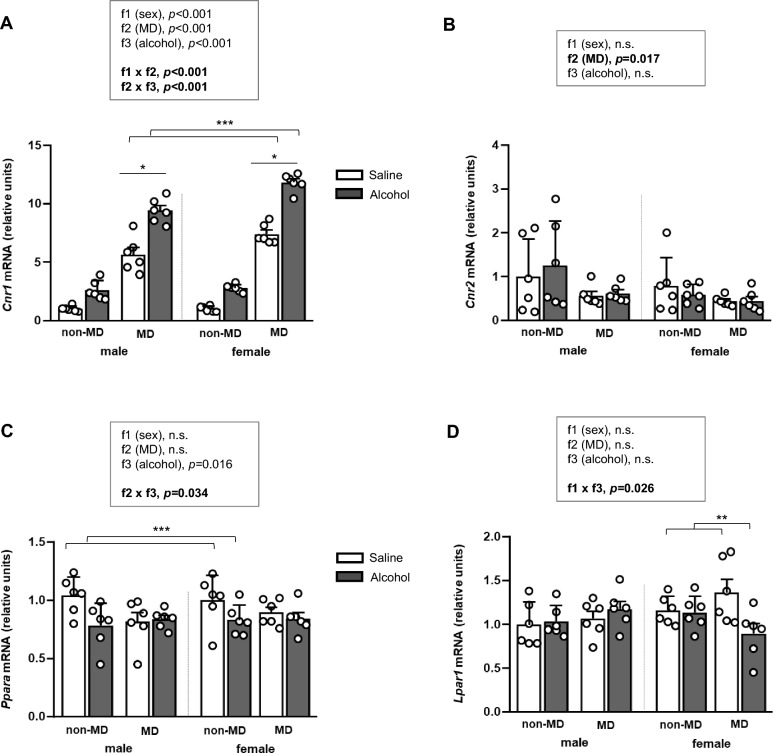
Effects of maternal deprivation, adolescent alcohol exposure, and sex on cannabinoid and LPA receptor gene expression in the mPFC. Relative mRNA expression of (**A**) *Cnr1*; (**B**) *Cnr2*; (**C**) *Ppara*; and (**D**) *Lpar1.* Data are expressed as mean ± SEM. Gene expression levels were normalized to the housekeeping gene and expressed relative to the non-MD saline male group. Statistical analyses were performed using three-way ANOVA. *p*-values in bold denote significant main effects of factors or significant interaction. Symbols indicate significant effects: (*) denotes *p* < 0.05, comparing MD rats to non-MD rats in males or females; (**) denotes *p* < 0.05, comparing alcohol-treated rats to saline-treated rats in males or females; (***) denotes *p* < 0.05, comparing alcohol-treated rats to saline-treated rats in the MD or non-MD groups

The mRNA expression of genes encoding ECS-related and LPA receptors (*Cnr1, Cnr2, Ppara*, *Lpar1*) in the mPFC was analyzed by three-way ANOVA with sex (f1), MD (f2), and adolescent alcohol exposure (f3) as factors (Fig. 4). Complete ANOVA statistics, including non-significant main effects and interactions, together with effect size estimates (ηp^2^), are provided in Supplementary Table S5.

#### mRNA expression of Cnr1

Cnr1 mRNA expression was significantly influenced by sex, MD, and adolescent alcohol exposure, and significant two-way interactions were also detected (Fig. 4A). A significant sex × MD interaction was observed [*F* (1,40) = 17.63, *p* < 0.001], indicating higher *Cnr1* expression in MD rats compared to non-MD rats, with this effect being more pronounced in females than in males. In addition, a significant MD × alcohol interaction was observed [*F* (1,40) = 25.84, *p* < 0.001], showing that alcohol-treated rats had higher *Cnr1* expression than saline-treated rats within the MD group, but not within the non-MD group.

#### mRNA expression of Cnr2

The analysis revealed only a significant main effect of MD [*F* (1,40) = 6.21, *p* = 0.017], with MD rats showing lower *Cnr2* expression than non-MD rats (Fig. 4B).

#### mRNA expression of Ppara

*Ppara* mRNA expression showed a significant main effect of adolescent alcohol exposure, along with a significant MD × alcohol interaction [*F* (1,40) = 4.80, *p* = 0.034]. Specifically, alcohol-treated rats had lower *Ppara* expression than saline-treated rats within the non-MD group, but not within the MD group (Fig. 4C).

#### mRNA expression of Lpar1

For *Lpar1*, the three-way ANOVA revealed a significant sex × alcohol interaction [*F* (1,40) = 5.39, *p* = 0.026], with alcohol-treated females exhibiting lower *Lpar1* expression than saline-treated females, but no differences were observed in males (Fig. 4D).

### Effects of MD, adolescent alcohol exposure, and sex on the mRNA expression of genes encoding enzymes involved in the ECS and LPA signaling in the mPFC

**Fig. 5:**
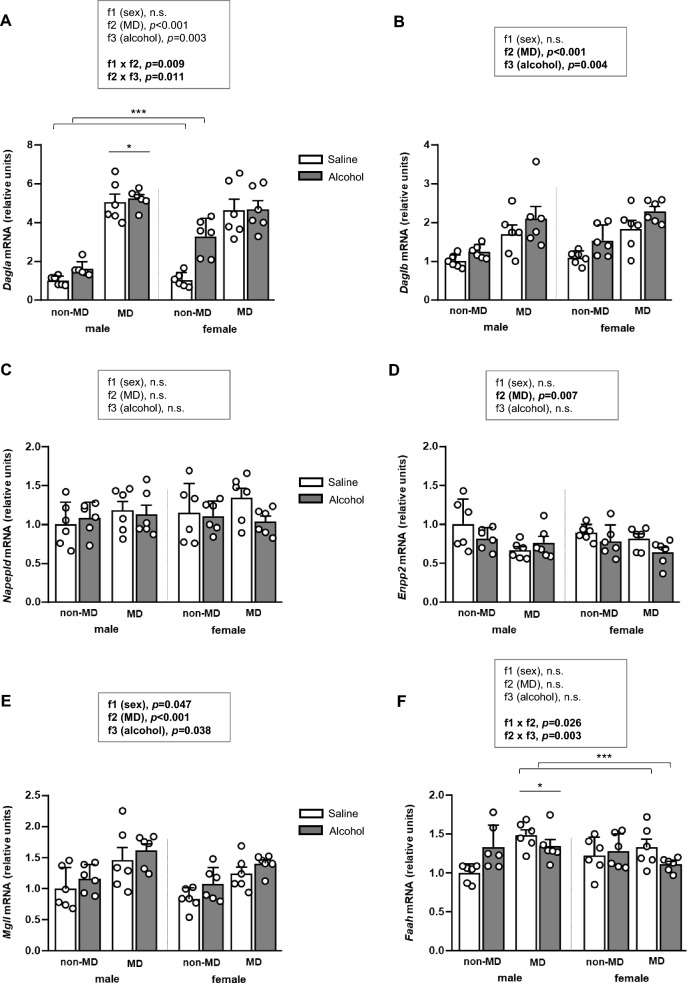
Effects of maternal deprivation, adolescent alcohol exposure, and sex on endocannabinoid- and LPA-related enzyme gene expression in the mPFC. Relative mRNA expression of (**A**) *Dagla*; (**B**) *Daglb*; (**C**) *Napepld*; (**D**) *Enpp2;* (**E**) *Mgll*; and (**F**) *Faah.* Data are expressed as mean ± SEM. Gene expression levels were normalized to the housekeeping gene and expressed relative to the non-MD saline male group. Statistical analyses were performed using three-way ANOVA. *p*-values in bold denote significant main effects of factors or significant interaction. Symbols indicate significant effects: (*) denotes *p* < 0.05, comparing MD rats to non-MD rats in males or females; (***) denotes *p* < 0.05, comparing alcohol-treated rats to saline-treated rats in the MD or non-MD groups

The mRNA expression of genes encoding enzymes involved in the synthesis and degradation of monoacylglycerols, N-acylethanolamines, and LPA in the mPFC was analyzed by three-way ANOVA with sex (f1), MD (f2), and adolescent alcohol exposure (f3) as factors (Fig. 5). Complete ANOVA statistics, including non-significant main effects and interactions, together with effect size estimates (ηp^2^), are provided in Supplementary Table S6.

#### mRNA expression of Dagla

There were significant main effects of MD and adolescent alcohol exposure on *Dagla* mRNA expression, along with significant two-way interactions (Fig. 5A). A significant sex × MD interaction was observed [*F* (1,40) = 7.62, *p* = 0.009], with MD males exhibiting higher *Dagla* expression compared to non-MD males, whereas no significant differences were observed in females. A significant MD × alcohol interaction was also observed [*F* (1,40) = 7.03, *p* = 0.011], indicating that alcohol-treated rats had higher *Dagla* expression than saline-treated rats within the non-MD group, but not within the MD group.

#### mRNA expression of Daglb

For *Daglb* mRNA expression, the analysis revealed a significant main effect of MD [*F* (1,40) = 36.97, *p* < 0.001], with MD rats exhibiting higher *Daglb* expression than non-MD rats (Fig. 5B). Additionally, a significant main effect of adolescent alcohol exposure was observed [*F* (1,40) = 9.37, *p* = 0.004], indicating higher *Daglb* expression in alcohol-treated rats compared to saline-treated rats.

#### mRNA expression of Napepld

*Napepld* mRNA expression showed no significant main effects or interactions for sex, MD, or adolescent alcohol exposure (Fig. 5C).

#### mRNA expression of Enpp2

The analysis revealed only a significant main effect of MD on *Enpp2* mRNA expression [*F* (1,40) = 8.12, *p* = 0.007], with MD rats showing lower *Enpp2* expression than non-MD rats (Fig. 5D).

#### mRNA expression of Mgll

A significant main effect of sex was observed for *Mgll* mRNA expression [*F* (1,40) = 4.19, *p* = 0.047], with females showing lower *Mgll* expression than males. There was also a significant main effect of MD on *Mgll* expression [*F* (1,40) = 23.91, *p* < 0.001], with MD rats exhibiting higher *Mgll* expression than non-MD rats. Additionally, a significant main effect of adolescent alcohol exposure was found [*F* (1,40) = 4.60, *p* = 0.038], indicating higher *Mgll* expression in alcohol-treated rats compared to saline-treated rats (Fig. 5E).

#### mRNA expression of Faah

Finally, the three-way ANOVA revealed significant interactions on *Faah* mRNA expression (Fig. 5F). Specifically, a significant sex × MD interaction was detected [*F* (1,40) = 5.35, *p* = 0.026], with post hoc comparisons indicating higher *Faah* expression in MD males compared to non-MD males, whereas no differences were observed in females. A significant MD × alcohol interaction was also observed [*F* (1,40) = 9.78, *p* = 0.003], showing that alcohol-treated rats had lower *Faah* expression than saline-treated rats within the MD group, but not within the non-MD group.

## Discussion

The present study demonstrates that early-life stress induced by MD interacts with adolescent alcohol exposure to produce persistent, sex-dependent changes in behavior, lipid signaling, and gene expression within the mPFC. Firstly, MD induces enduring metabolic alterations, including reduced body weight gain, and reveals sex-specific effects on alcohol pharmacokinetics, with MD females exhibiting higher BAC than non-MD females. Secondly, behavioral alterations exhibit clear sexual dimorphism: MD increases active stress-coping behavior in males while decreasing escape behavior in females, and promotes greater open-arm exploration in the EPM, independent of alcohol exposure. Third, adolescent alcohol exposure elevates circulating corticosterone levels; in contrast, MD and sex selectively alter the profile of circulating lipid mediators, including monoacylglycerols, N-acylethanolamines, and LPA, with pronounced sex-dependent interaction effects. Finally, MD and alcohol exposure differentially modulate the expression of cannabinoid and LPA receptors, as well as lipid-metabolizing enzymes in the mPFC, revealing distinct molecular patterns in males and females. Collectively, these findings suggest that the long-term consequences of early-life stress and adolescent alcohol exposure are sex-dependent and involve coordinated adaptations at both peripheral and central levels.

Consistent with previous reports, MD induced a significant reduction in body weight immediately after the deprivation episode, followed by a sustained attenuation of weight gain one week later, irrespective of sex. These findings confirm the metabolic and physiological impact of a single episode of MD during a critical postnatal developmental period [23, 50]. During adolescent alcohol exposure, male rats exhibited greater body weight gain and food intake compared to females [18, 23]. Notably, MD animals demonstrated increased food intake during adolescent alcohol exposure, despite earlier studies describing decreased food intake following early-life stress [51, 52]. This apparent contradiction may be clarified by the experimental context, as food intake was measured during repeated alcohol exposure, a condition known to disrupt metabolic signaling and energy homeostasis [53]. Consequently, the increased food intake observed in MD rats may reflect a compensatory response to alcohol-induced metabolic alterations. These findings further imply that early-life stress may modify metabolic adaptations to subsequent environmental challenges such as alcohol exposure [54].

In accordance with our prior studies, adolescent alcohol exposure resulted in increased BAC over time, indicating cumulative effects of repeated exposure [18, 20]. Importantly, MD selectively increased BAC in female rats, suggesting that early-life stress may alter alcohol metabolism or absorption in a sex-specific manner [55, 56]. This increase in BAC may be associated with greater vulnerability to alcohol-induced neurobiological alterations and increased sensitivity to alcohol-related effects [16, 57].

Behavioral analyses revealed that MD induced sex-dependent adaptations in stress-coping behavior. Although total immobility time in the FST was not altered, MD males exhibited increased escape behavior, whereas MD females showed reduced active coping responses. The absence of changes in immobility does not necessarily indicate a lack of effect on stress responses, as the FST is increasingly considered a measure of coping strategy rather than behavioral despair [58, 59]. Moreover, adolescent alcohol exposure increased escape behavior in MD animals, suggesting that adolescent alcohol may potentiate stress responsivity in individuals previously exposed to early-life adversity [60].

Similarly, anxiety-like behavior assessed in the EPM also exhibited sex-dependent effects. Consistent with previous reports, female rats displayed greater open-arm exploration than males [61, 62], as reflected by increased OTR, greater distance traveled in the open arms, and a higher number of open-arm entries, together with reduced time spent in the closed arms. Furthermore, MD rats showed increased open-arm exploration compared to non-MD rats, regardless of adolescent alcohol exposure. This pattern may not solely indicate reduced anxiety but could represent adaptive modifications in stress responsiveness following early-life adversity [63]. Considering that the EPM was conducted several days after the FST, which itself acts as a stressor [64], these findings likely reflect the combined impact of early-life stress history and recent stress exposure. Moreover, no significant differences were observed in total distance traveled or time spent in the center zone, suggesting that the observed changes in open-arm exploration were not attributable to generalized alterations in locomotor activity or freezing-like behavior. Additionally, behavioral adaptations were not accompanied by baseline alterations in HPA axis activity. Plasma corticosterone levels were elevated following adolescent alcohol exposure, in line with alcohol-induced activation of the HPA axis [19, 20]. Additionally, it should be considered that alcohol exposure was administered via intragastric gavage, which may itself represent an additional source of stress. However, control animals received saline administration following the same handling and gavage procedure, thereby controlling for potential stress associated with repeated administration. Future studies using voluntary alcohol consumption paradigms may help further distinguish the pharmacological effects of alcohol from those potentially associated with the administration procedure. In contrast, MD did not significantly affect corticosterone levels at the measured time point. Although early-life stress is known to produce enduring modifications in HPA axis regulation [5], the absence of MD-related differences in corticosterone in this study may be attributable to the experimental context, wherein hormone levels were measured after exposure to an acute behavioral stressor. Importantly, these behavioral adaptations occurred in parallel with alterations in lipid signaling pathways. Specifically, endocannabinoid-related lipids and LPA are recognized modulators of stress responsivity, emotional behavior, and synaptic plasticity [28, 31, 39, 40], suggesting coordinated stress-related adaptations at both peripheral and central levels.

Consistent with this notion, our findings demonstrated that MD and adolescent alcohol exposure also produced sex-dependent alterations in circulating endocannabinoid-related lipid mediators and LPA. Previous research has shown that adolescent restraint stress increases plasma lipid levels, particularly monoacylglycerols, with these effects persisting for several weeks post-exposure [20]. In the current study, MD resulted in a selective, sex-dependent reorganization of plasma lipid profiles. Specifically, MD males exhibited increased levels of certain monoacylglycerols, whereas MD females displayed consistent reductions in both monoacylglycerols and their corresponding N-acylethanolamines across multiple fatty acid classes, consistent with previous evidence indicating sex-dependent regulation of endocannabinoid signaling under stress conditions [65]. Although the physiological implications of these alterations remain to be fully elucidated, these results suggest that MD induces long-lasting modifications in peripheral endocannabinoid signaling, potentially influencing emotional regulation [66]. In agreement with our previous research involving male rats subjected to an identical alcohol exposure protocol [20], adolescent alcohol exposure did not produce a consistent global effect on plasma levels of endocannabinoid-related lipids. Instead, alcohol exposure appeared to modulate lipid signaling in a context-dependent manner, as reflected by specific effects and interactions with MD observed for individual lipid species, including 2-LG, LEA, 2-OG, and 2-AG. This pattern is consistent with the previous findings showing that alcohol exposure can alter or even blunt stress-induced changes in endocannabinoid levels [20]. Nonetheless, a pronounced main effect of sex was observed, with females exhibiting higher baseline levels than males, particularly for monoacylglycerols 2-LG and 2-OG. While prior clinical investigations have not reported similar basal sex differences in circulating endocannabinoids [67], such discrepancies may stem from interspecies variations or methodological differences [68]. Furthermore, the pre-exposure to the FST in the present study may have contributed to these sex differences, as acute stress can increase circulating endocannabinoids [69]. The reduced magnitude of this apparent increase in females subjected to MD and/or adolescent alcohol suggests that early-life adversity and alcohol intake may blunt stress-induced activation of the peripheral endocannabinoid system [70].

Regarding LPA signaling, we observed a distinct sexual dimorphism in circulating LPA levels, with females exhibiting higher plasma concentrations than males, consistent with previous clinical studies [43, 45, 71, 72]. Furthermore, MD decreased plasma LPA levels in both sexes, indicating that early-life stress results in persistent modifications in peripheral LPA signaling. These effects were only partially related to changes in the primary enzyme responsible for LPA synthesis, as autotaxin levels were selectively elevated by adolescent alcohol exposure only in non-MD animals. This implies that the alterations in circulating LPA associated with MD and sex may originate from mechanisms independent of autotaxin, such as altered LPA clearance, substrate availability, or tissue-specific release [73, 74]. Additionally, the alcohol-induced increase in autotaxin observed exclusively in non-MD rats may reflect a stress-sensitive regulation of LPA biosynthesis disrupted by early-life adversity. Collectively, these findings indicate that peripheral LPA signaling is differentially modulated by early-life stress, sex, and adolescent alcohol exposure.

In addition to peripheral lipid alterations, our findings indicate that MD and adolescent alcohol exposure induce a significant and sex-dependent reorganization of endocannabinoid- and LPA-related signaling within the mPFC. These observations align with prior evidence implicating these systems in stress responses and alcohol-related neuroadaptations [28, 30, 40, 75]. Notably, these central molecular adaptations in the mPFC occurred in parallel with peripheral lipid alterations and behavioral adaptations, suggesting coordinated responses across stress-related systems. Considering the critical role of the mPFC in integrating stress-related signals and directing adaptive responses [76], the coordinated changes observed in lipid signaling pathways and gene expression may underlie the sex-dependent behavioral phenotypes identified herein. Consistent with previous research demonstrating that repeated alcohol exposure increases CB_1_ receptor expression in prefrontal regions [19], we observed an upregulation of *Cnr1* expression following adolescent alcohol exposure. Importantly, MD alone also increased mRNA expression of this receptor, and this effect was further amplified in animals exposed to both MD and alcohol, indicating that early-life stress may enhance the responsiveness of CB_1_-mediated signaling to subsequent adolescent challenges. Given the critical role of these receptors in regulating synaptic plasticity, stress responsivity, and emotional behavior in the PFC [77, 78], such adaptations may contribute to the mechanisms through which early adversity influences vulnerability to alcohol-induced neurobiological alterations. By contrast, *Cnr2* expression was decreased following MD. Considering the well-documented role of CB_2_ receptors in modulating neuroimmune responses and microglial activity [for review see [79]], this reduction may contribute to altered neuroimmune regulation subsequent to early-life stress. Additional changes were observed in other lipid-related receptors. Thus, *Ppara* expression was decreased in non-MD alcohol-exposed rats, suggesting that adolescent alcohol exposure may influence transcriptional pathways involved in lipid metabolism and inflammatory regulation within the mPFC. In contrast, *Lpar1* expression was reduced in female rats following adolescent alcohol exposure, consistent with a sex-dependent effect. Although this reduction appeared more pronounced in animals exposed to both MD and alcohol, no significant interaction involving MD was detected. These findings suggest a sex-dependent modulation of LPA signaling by alcohol exposure, consistent with previous clinical observations reporting sexual dimorphism in circulating LPA species in patients with alcohol use disorder [43, 44, 71]. Changes in receptor expression were accompanied by alterations in enzymes involved in endocannabinoid synthesis and degradation within the mPFC. Specifically, MD and adolescent alcohol exposure modulated the expression of enzymes responsible for 2-AG synthesis and degradation, implying a reorganization of endocannabinoid tone in this brain region. These findings are consistent with previous research indicating that early-life stress increases endocannabinoid-related gene expression in the PFC [80] and that repeated alcohol exposure likewise influences endocannabinoid signaling in this region [19]. Furthermore, the absence of change in *Napepld* expression suggests that these adaptations may selectively involve monoacylglycerol pathways rather than N-acylethanolamine metabolism. Additionally, the reduction of *Enpp2* expression in MD-exposed rats implies that early-life stress may also impact LPA biosynthesis within the mPFC, supporting the idea that developmental stress broadly reorganizes lipid signaling networks in this brain region.

Importantly, the present study revealed pronounced sex-dependent differences across behavioral, peripheral lipid, and central molecular measures, suggesting that males and females show different neurobiological adaptations to early-life stress and adolescent alcohol exposure. These differences likely reflect sex-specific developmental trajectories occurring during sensitive neurodevelopmental periods, including differential maturation of prefrontal-limbic circuitry, HPA axis regulation, and lipid signaling systems [81–83]. Given that both the early postnatal period and adolescence are characterized by profound endocrine and neurobiological transitions, early-life adversity and alcohol exposure may differentially interfere with ongoing maturation processes in males and females [84]. Such sex-dependent programming effects may contribute to differential vulnerability or adaptive responses to stress-related psychopathology later in life.

However, several considerations should be taken into account when interpreting these results. First, the use of the FST prior to anxiety-like assessments may have impacted subsequent behavioral or molecular measures. While this possibility cannot be entirely excluded, all animals were subjected to the same testing sequence, thereby permitting valid comparisons across experimental conditions. Furthermore, the temporal separation between behavioral assessments and tissue collection reduces the likelihood that acute stress effects solely account for the observed molecular changes. Second, although animals were distributed across litters to minimize potential bias, litter was not included as a factor in the statistical analysis, which should be considered when interpreting the findings. Third, the MD paradigm employed in this study involves not only disruption of maternal care, but also additional physiological stressors associated with prolonged separation, including temporary fasting and absence of external heating. Therefore, some of the long-term effects observed may reflect the combined impact of maternal separation and these physiological challenges. Since acute physiological parameters such as body temperature or hydration status were not monitored during the deprivation period, their potential contribution cannot be excluded. Fourth, the study focused on transcriptional modifications without concurrent assessment of protein levels or functional activity, limiting the ability to infer the downstream consequences of these molecular adaptations. Furthermore, although peripheral lipid alterations and central molecular adaptations were observed in parallel, the present study does not establish direct mechanistic coupling between circulating markers and mPFC signaling changes. Therefore, causal relationships between peripheral and central alterations cannot be inferred from the current data. Finally, although the present study focused on the mPFC due to its central role in integrating stress-related signals and exerting top-down control over subcortical regions, other brain areas such as the ventral hippocampus, amygdala, nucleus accumbens, and hypothalamus are also critically involved in stress and emotional regulation. Future studies should extend these findings to additional regions to provide a more comprehensive understanding of the neurocircuitry underlying the observed effects.

## Perspectives and significance

The present study provides evidence that early-life stress induced by MD programs long-lasting, sex-specific adaptations in behavioral coping strategies and lipid-mediated stress signaling. Adolescent alcohol exposure serves as an additional challenge that reveals or amplifies these programmed differences, particularly within endocannabinoid and LPA signaling systems. The pronounced sexual dimorphism observed across behavioral, peripheral, and central alterations further emphasizes the importance of considering sex as a critical biological variable in investigations addressing the long-term consequences of early-life adversity. By integrating behavioral analyses with peripheral lipid profiles and mPFC gene expression, this study supports the involvement of lipid signaling pathways in the long-term adaptations associated with early adversity and adolescent experiences that may contribute to vulnerability to stress-related psychopathology. These findings contribute to an expanding body of evidence positioning the endocannabinoid system and LPA signaling as central regulators of stress adaptation and emotional behavior, and they suggest that dysregulation within these pathways may constitute a mechanistic substrate through which early-life experiences influence subsequent susceptibility to neuropsychiatric conditions.

## Supplementary Information

Supplementary Material 1

Supplementary Material 2

Supplementary Material 3

Supplementary Material 4

Supplementary Material 5

Supplementary Material 6

Supplementary Material 7

## Data Availability

The datasets generated and/or analyzed during the current study are available from the corresponding author on reasonable request.

## References

[1] Agorastos A, Pervanidou P, Chrousos GP, Baker DG. Developmental trajectories of early life stress and trauma: a narrative review on neurobiological aspects beyond stress system dysregulation. Front Psychiatry. 2019;10:118. 30914979 10.3389/fpsyt.2019.00118 PMC6421311

[2] Fenton MC, Geier T, Keyes K, Skodol AE, Grant BF, Hasin DS. Combined role of childhood maltreatment, family history, and gender in the risk for alcohol dependence. Psychol Med. 2013;43(5):1045–57. 22883538 10.1017/S0033291712001729 PMC3767412

[3] Holz NE, Berhe O, Sacu S, Schwarz E, Tesarz J, Heim CM, et al. Early social adversity, altered brain functional connectivity, and mental health. Biol Psychiatry. 2023;93(5):430–41. 36581495 10.1016/j.biopsych.2022.10.019

[4] Strine TW, Dube SR, Edwards VJ, Prehn AW, Rasmussen S, Wagenfeld M, et al. Associations between adverse childhood experiences, psychological distress, and adult alcohol problems. Am J Health Behav. 2012;36(3):408–23. 22370441 10.5993/AJHB.36.3.11

[5] Nishi M. Effects of early-life stress on the brain and behaviors: implications of early maternal separation in Rodents. Int J Mol Sci. 2020;21(19):7212. 33003605 10.3390/ijms21197212 PMC7584021

[6] Aisa B, Tordera R, Lasheras B, Del Rio J, Ramirez MJ. Effects of maternal separation on hypothalamic-pituitary-adrenal responses, cognition and vulnerability to stress in adult female rats. Neuroscience. 2008;154(4):1218–26. 18554808 10.1016/j.neuroscience.2008.05.011

[7] Lippmann M, Bress A, Nemeroff CB, Plotsky PM, Monteggia LM. Long-term behavioural and molecular alterations associated with maternal separation in rats. Eur J Neurosci. 2007;25(10):3091–8. 17561822 10.1111/j.1460-9568.2007.05522.x

[8] Poleksic J, Aksic M, Kapor S, Aleksic D, Stojkovic T, Radovic M, et al. Effects of maternal deprivation on the prefrontal cortex of male rats: cellular, neurochemical, and behavioral outcomes. Front Behav Neurosci. 2021;15:666547. 34819843 10.3389/fnbeh.2021.666547 PMC8606589

[9] Aleksic D, Poleksic J, Agatonovic G, Djulejic V, Vulovic M, Aksic M, et al. The long-term effects of maternal deprivation on the number and size of inhibitory interneurons in the rat amygdala and nucleus accumbens. Front Neurosci. 2023;17:1187758. 37434764 10.3389/fnins.2023.1187758 PMC10330809

[10] Miragaia AS, de Oliveira Wertheimer GS, Consoli AC, Cabbia R, Longo BM, Girardi CEN, et al. Maternal deprivation increases anxiety- and depressive-like behaviors in an age-dependent fashion and reduces neuropeptide Y expression in the amygdala and hippocampus of male and female young adult rats. Front Behav Neurosci. 2018;12:159. 30131681 10.3389/fnbeh.2018.00159 PMC6090069

[11] Delavari F, Sheibani V, Esmaeili-Mahani S, Nakhaee N. Maternal separation and the risk of drug abuse in later life. Addict Health. 2016;8(2):107–14. 27882208 PMC5115644

[12] Marco EM, Valero M, de la Serna O, Aisa B, Borcel E, Ramirez MJ, et al. Maternal deprivation effects on brain plasticity and recognition memory in adolescent male and female rats. Neuropharmacology. 2013;68:223–31. 22939999 10.1016/j.neuropharm.2012.08.014

[13] Oomen CA, Girardi CE, Cahyadi R, Verbeek EC, Krugers H, Joels M, et al. Opposite effects of early maternal deprivation on neurogenesis in male versus female rats. PLoS ONE. 2009;4(1):e3675. 19180242 10.1371/journal.pone.0003675 PMC2629844

[14] Xu H, Ye Y, Hao Y, Shi F, Yan Z, Yuan G, et al. Sex differences in associations between maternal deprivation and alterations in hippocampal calcium-binding proteins and cognitive functions in rats. Behav Brain Funct. 2018;14(1):10. 29759084 10.1186/s12993-018-0142-y PMC5952636

[15] Miller JG, Chahal R, Gotlib IH. Early life stress and neurodevelopment in adolescence: implications for risk and adaptation. Curr Top Behav Neurosci. 2022;54:313–39. 35290658 10.1007/7854_2022_302

[16] Crews FT, Robinson DL, Chandler LJ, Ehlers CL, Mulholland PJ, Pandey SC, et al. Mechanisms of persistent neurobiological changes following adolescent alcohol exposure: NADIA consortium findings. Alcohol Clin Exp Res. 2019;43(9):1806–22. 31335972 10.1111/acer.14154 PMC6758927

[17] Spear LP. Effects of adolescent alcohol consumption on the brain and behaviour. Nat Rev Neurosci. 2018;19(4):197–214. 29467469 10.1038/nrn.2018.10

[18] Gobbi C, Sanchez-Marin L, Flores-Lopez M, Medina-Vera D, Pavon-Moron FJ, de Fonseca RF, et al. Sex-dependent effects of acute stress and alcohol exposure during adolescence on mRNA expression of brain signaling systems involved in reward and stress responses in young adult rats. Biol Sex Differ. 2024;15(1):75. 39327618 10.1186/s13293-024-00649-5 PMC11426001

[19] Sanchez-Marin L, Flores-Lopez M, Gavito AL, Suarez J, Pavon-Moron FJ, de Fonseca FR, et al. Repeated restraint stress and binge alcohol during adolescence induce long-term effects on anxiety-like behavior and the expression of the endocannabinoid system in male rats. Biomedicines. 2022. 10.3390/biomedicines10030593. 35327395 10.3390/biomedicines10030593 PMC8945821

[20] Sanchez-Marin L, Flores-Lopez M, Pastor A, Gavito AL, Suarez J, de la Torre R, et al. Acute stress and alcohol exposure during adolescence result in an anxious phenotype in adulthood: Role of altered glutamate/endocannabinoid transmission mechanisms. Prog Neuropsychopharmacol Biol Psychiatry. 2022;113:110460. 34695542 10.1016/j.pnpbp.2021.110460

[21] Sanchez-Marin L, Gavito AL, Decara J, Pastor A, Castilla-Ortega E, Suarez J, et al. Impact of intermittent voluntary ethanol consumption during adolescence on the expression of endocannabinoid system and neuroinflammatory mediators. Eur Neuropsychopharmacol. 2020;33:126–38. 32057593 10.1016/j.euroneuro.2020.01.012

[22] Llorente R, Miguel-Blanco C, Aisa B, Lachize S, Borcel E, Meijer OC, et al. Long term sex-dependent psychoneuroendocrine effects of maternal deprivation and juvenile unpredictable stress in rats. J Neuroendocrinol. 2011;23(4):329–44. 21219484 10.1111/j.1365-2826.2011.02109.x

[23] Penasco S, Mela V, Lopez-Moreno JA, Viveros MP, Marco EM. Early maternal deprivation enhances voluntary alcohol intake induced by exposure to stressful events later in life. Neural Plast. 2015;2015:342761. 25821601 10.1155/2015/342761 PMC4363574

[24] Gildawie KR, Ryll LM, Hexter JC, Peterzell S, Valentine AA, Brenhouse HC. A two-hit adversity model in developing rats reveals sex-specific impacts on prefrontal cortex structure and behavior. Dev Cogn Neurosci. 2021;48:100924. 33515957 10.1016/j.dcn.2021.100924 PMC7847967

[25] Daskalakis NP, Bagot RC, Parker KJ, Vinkers CH, de Kloet ER. The three-hit concept of vulnerability and resilience: toward understanding adaptation to early-life adversity outcome. Psychoneuroendocrinology. 2013;38(9):1858–73. 23838101 10.1016/j.psyneuen.2013.06.008 PMC3773020

[26] Estes ML, McAllister AK. Maternal immune activation: implications for neuropsychiatric disorders. Science. 2016;353(6301):772–7. 27540164 10.1126/science.aag3194 PMC5650490

[27] Brenhouse HC, Andersen SL. Developmental trajectories during adolescence in males and females: a cross-species understanding of underlying brain changes. Neurosci Biobehav Rev. 2011;35(8):1687–703. 21600919 10.1016/j.neubiorev.2011.04.013 PMC3134153

[28] Morena M, Patel S, Bains JS, Hill MN. Neurobiological interactions between stress and the endocannabinoid system. Neuropsychopharmacology. 2016;41(1):80–102. 26068727 10.1038/npp.2015.166 PMC4677118

[29] Basavarajappa BS, Joshi V, Shivakumar M, Subbanna S. Distinct functions of endogenous cannabinoid system in alcohol abuse disorders. Br J Pharmacol. 2019;176(17):3085–109. 31265740 10.1111/bph.14780 PMC6692638

[30] Kunos G. Interactions between alcohol and the endocannabinoid system. Alcohol Clin Exp Res. 2020;44(4):790–805. 32056226 10.1111/acer.14306 PMC8760843

[31] Micale V, Drago F. Endocannabinoid system, stress and HPA axis. Eur J Pharmacol. 2018;834:230–9. 30036537 10.1016/j.ejphar.2018.07.039

[32] Lowe H, Toyang N, Steele B, Bryant J, Ngwa W. The endocannabinoid system: a potential target for the treatment of various diseases. Int J Mol Sci. 2021. 10.3390/ijms22179472. 34502379 10.3390/ijms22179472 PMC8430969

[33] Serrano A, Natividad LA. Alcohol-endocannabinoid interactions: implications for addiction-related behavioral processes. Alcohol Res. 2022;42(1):09. 35655710 10.35946/arcr.v42.1.09 PMC9132373

[34] Serrano A, Parsons LH. Endocannabinoid influence in drug reinforcement, dependence and addiction-related behaviors. Pharmacol Ther. 2011;132(3):215–41. 21798285 10.1016/j.pharmthera.2011.06.005 PMC3209522

[35] Zhao P, Abood ME. GPR55 and GPR35 and their relationship to cannabinoid and lysophospholipid receptors. Life Sci. 2013;92(8–9):453–7. 22820167 10.1016/j.lfs.2012.06.039

[36] Shim YH, Lin CH, Strickland KP. The purification and properties of monoacylglycerol kinase from bovine brain. Biochem Cell Biol. 1989;67(4–5):233–41. 2550036 10.1139/o89-035

[37] Nakane S, Oka S, Arai S, Waku K, Ishima Y, Tokumura A, et al. 2-Arachidonoyl-sn-glycero-3-phosphate, an arachidonic acid-containing lysophosphatidic acid: occurrence and rapid enzymatic conversion to 2-arachidonoyl-sn-glycerol, a cannabinoid receptor ligand, in rat brain. Arch Biochem Biophys. 2002;402(1):51–8. 12051682 10.1016/S0003-9861(02)00038-3

[38] Gonzalez de San Roman E, Manuel I, Ledent C, Chun J, de Fonseca RF, Estivill-Torrus G, et al. CB(1) and LPA(1) receptors relationship in the mouse central nervous system. Front Mol Neurosci. 2019;12:223. 31607860 10.3389/fnmol.2019.00223 PMC6761275

[39] Yung YC, Stoddard NC, Mirendil H, Chun J. Lysophosphatidic acid signaling in the nervous system. Neuron. 2015;85(4):669–82. 25695267 10.1016/j.neuron.2015.01.009 PMC4400838

[40] Moreno-Fernandez RD, Tabbai S, Castilla-Ortega E, Perez-Martin M, Estivill-Torrus G, de Fonseca RF, et al. Stress, depression, resilience and ageing: a role for the LPA-LPA1 pathway. Curr Neuropharmacol. 2018;16(3):271–83. 28699486 10.2174/1570159X15666170710200352 PMC5843979

[41] Tabbai S, Moreno-Fernandez RD, Zambrana-Infantes E, Nieto-Quero A, Chun J, Garcia-Fernandez M, et al. Effects of the LPA(1) Receptor Deficiency and Stress on the Hippocampal LPA Species in Mice. Front Mol Neurosci. 2019;12:146. 31244601 10.3389/fnmol.2019.00146 PMC6580287

[42] Castro-Zavala A, Boonen K, Sanchez-Marin L, Roberto M, Pavon-Moron FJ, de Fonseca RF, et al. Fluoxetine modulates both endocannabinoid and lysophosphatidic acid pathways in a region-specific manner during alcohol withdrawal in male rats. Neuropharmacology. 2026;284:110773. 41271090 10.1016/j.neuropharm.2025.110773

[43] Flores-Lopez M, Garcia-Marchena N, Araos P, Requena-Ocana N, Porras-Perales O, Torres-Galvan S, et al. Sex differences in plasma lysophosphatidic acid species in patients with alcohol and cocaine use disorders. Brain Sci. 2022. 10.3390/brainsci12050588. 35624975 10.3390/brainsci12050588 PMC9139721

[44] Flores-Lopez M, Garcia-Marchena N, Pavon FJ, Lara E, Porras-Perales O, Araos P, et al. Plasma concentrations of lysophosphatidic acid and autotaxin in abstinent patients with alcohol use disorder and comorbid liver disease. Biomedicines. 2021. 10.3390/biomedicines9091207. 34572393 10.3390/biomedicines9091207 PMC8469650

[45] Flores-Lopez M, Garcia-Marchena N, Pavon-Moron FJ, Requena-Ocana N, Sanchez-Marin L, Martin-Chaves L, et al. Plasma concentrations of lysophosphatidic acid and the expression of its receptors in peripheral blood mononuclear cells are altered in patients with cocaine use disorders. Transl Psychiatry. 2023;13(1):215. 37344453 10.1038/s41398-023-02523-1 PMC10284796

[46] San Felipe D, Martin-Sanchez B, Zekri-Nechar K, Moya M, Llorente R, Zamorano-Leon JJ, et al. Consequences of early maternal deprivation on neuroinflammation and mitochondrial dynamics in the central nervous system of male and female rats. Biology. 2024. 10.3390/biology13121011. 39765678 10.3390/biology13121011 PMC11672930

[47] Verheul-Campos J, Sanchez-Marin L, Lopez Y, Gavito AL, Grandes P, Serrano P, et al. Prior restraint stress counteracts memory deficits associated with adolescent alcohol exposure by targeting both the hippocampal endocannabinoid and glutamatergic systems. Drug Alcohol Depend. 2025;276:112891. 40975942 10.1016/j.drugalcdep.2025.112891

[48] Paxinos G, Watson C. The Rat Brain in Stereotaxic Coordinates. New York: Academic Press, Spiral Bound; 1998.

[49] Pastor A, Farre M, Fito M, Fernandez-Aranda F, de la Torre R. Analysis of ECs and related compounds in plasma: artifactual isomerization and ex vivo enzymatic generation of 2-MGs. J Lipid Res. 2014;55(5):966–77. 24610889 10.1194/jlr.D043794 PMC3995474

[50] Viveros MP, Llorente R, Diaz F, Romero-Zerbo SY, Bermudez-Silva FJ, de Fonseca FR, et al. Maternal deprivation has sexually dimorphic long-term effects on hypothalamic cell-turnover, body weight and circulating hormone levels. Horm Behav. 2010;58(5):808–19. 20708008 10.1016/j.yhbeh.2010.08.003

[51] Mela V, Llorente-Berzal A, Diaz F, Argente J, Viveros MP, Chowen JA. Maternal deprivation exacerbates the response to a high fat diet in a sexually dimorphic manner. PLoS ONE. 2012;7(11):e48915. 23145019 10.1371/journal.pone.0048915 PMC3492147

[52] Wertheimer GS, Girardi CE, de Oliveira AS, Monteiro Longo B, Suchecki D. Maternal deprivation alters growth, food intake, and neuropeptide Y in the hypothalamus of adolescent male and female rats. Dev Psychobiol. 2016;58(8):1066–75. 27307308 10.1002/dev.21440

[53] Lee J, Lee JY, Kang H. Excessive alcohol consumption: a driver of metabolic dysfunction and inflammation. Front Toxicol. 2025;7:1670769. 41090150 10.3389/ftox.2025.1670769 PMC12515849

[54] Maniam J, Antoniadis C, Morris MJ. Early-life stress, HPA axis adaptation, and mechanisms contributing to later health outcomes. Front Endocrinol (Lausanne). 2014;5:73. 24860550 10.3389/fendo.2014.00073 PMC4026717

[55] Glover EJ, Khan F, Clayton-Stiglbauer K, Chandler LJ. Impact of sex, strain, and age on blood ethanol concentration and behavioral signs of intoxication during ethanol vapor exposure. Neuropharmacology. 2021;184:108393. 33221480 10.1016/j.neuropharm.2020.108393 PMC7855756

[56] Mezey E. Stress and ethanol metabolism. Alcohol Alcohol. 1998;33(3):310. 9632057 10.1093/oxfordjournals.alcalc.a008395

[57] McCaul ME, Roach D, Hasin DS, Weisner C, Chang G, Sinha R. Alcohol and women: a brief overview. Alcohol Clin Exp Res. 2019;43(5):774–9. 30779446 10.1111/acer.13985 PMC6502688

[58] de Kloet ER, Molendijk ML. Coping with the Forced Swim Stressor: towards understanding an adaptive mechanism. Neural Plast. 2016;2016:6503162. 27034848 10.1155/2016/6503162 PMC4806646

[59] Commons KG, Cholanians AB, Babb JA, Ehlinger DG. The Rodent Forced Swim Test measures stress-coping strategy, not depression-like behavior. ACS Chem Neurosci. 2017;8(5):955–60. 28287253 10.1021/acschemneuro.7b00042 PMC5518600

[60] Morningstar AR, Ledbury OSR, Yu AC, Sardar H, Rogers ET, Kandil IF, et al. Early life stress modulates behavioral sensitivity to alcohol and promotes escalation of alcohol drinking. bioRxiv. 2025.

[61] Borchers S, Krieger JP, Asker M, Maric I, Skibicka KP. Commonly-used rodent tests of anxiety-like behavior lack predictive validity for human sex differences. Psychoneuroendocrinology. 2022;141:105733. 35367714 10.1016/j.psyneuen.2022.105733

[62] Scholl JL, Afzal A, Fox LC, Watt MJ, Forster GL. Sex differences in anxiety-like behaviors in rats. Physiol Behav. 2019;211:112670. 31487491 10.1016/j.physbeh.2019.112670

[63] Hao Y, Niu Y, Shi F, Zhang L, Peng C, Yan Z, et al. A single 24 h maternal separation at PND 9 promotes behavioral resilience of female C57BL/6J mice and the possible role of hippocampal Homer1a. Heliyon. 2024;10(5):e27037. 38455582 10.1016/j.heliyon.2024.e27037 PMC10918190

[64] Molendijk ML, de Kloet ER. Coping with the forced swim stressor: current state-of-the-art. Behav Brain Res. 2019;364:1–10. 30738104 10.1016/j.bbr.2019.02.005

[65] Dow-Edwards D. Sex differences in the interactive effects of early life stress and the endocannabinoid system. Neurotoxicol Teratol. 2020;80:106893. 32437941 10.1016/j.ntt.2020.106893

[66] Hill MN, Miller GE, Ho WS, Gorzalka BB, Hillard CJ. Serum endocannabinoid content is altered in females with depressive disorders: a preliminary report. Pharmacopsychiatry. 2008;41(2):48–53. 18311684 10.1055/s-2007-993211 PMC3422568

[67] Flores-Lopez M, Herrera-Imbroda J, Requena-Ocana N, Garcia-Marchena N, Araos P, Verheul-Campos J, et al. Exploratory study on plasma Acylglycerol and Acylethanolamide dysregulation in substance use and attention-deficit/hyperactivity disorder: implications for novel biomarkers in dual diagnosis. Prog Neuropsychopharmacol Biol Psychiatry. 2025;138:111350. 40188983 10.1016/j.pnpbp.2025.111350

[68] Lawley S, Green A, Johnson C, Burton MD. Interspecies differences in the expression of cannabinoid receptors at the tissue and cellular levels. Neural Regen Res. 2025. 10.4103/NRR.NRR-D-25-00806 PMC13557655 41169214

[69] Dlugos A, Childs E, Stuhr KL, Hillard CJ, de Wit H. Acute stress increases circulating anandamide and other N-acylethanolamines in healthy humans. Neuropsychopharmacology. 2012;37(11):2416–27. 22763622 10.1038/npp.2012.100 PMC3442338

[70] Sanchez-Marin L, Canoluk B, Verheul-Campos J, Gavito A, Reviriego R, Pavon J, et al. Prior stress history shapes adolescent alcohol-induced transcriptional changes in striatal glutamatergic and endocannabinoid pathways. Adicciones. 2025;37(4):369–82. 41557451 10.20882/adicciones.2483

[71] Garcia-Marchena N, Pizarro N, Pavon FJ, Martinez-Huelamo M, Flores-Lopez M, Requena-Ocana N, et al. Potential association of plasma lysophosphatidic acid (LPA) species with cognitive impairment in abstinent alcohol use disorders outpatients. Sci Rep. 2020;10(1):17163. 33051508 10.1038/s41598-020-74155-0 PMC7555527

[72] Michalczyk A, Budkowska M, Dolegowska B, Chlubek D, Safranow K. Lysophosphatidic acid plasma concentrations in healthy subjects: circadian rhythm and associations with demographic, anthropometric and biochemical parameters. Lipids Health Dis. 2017;16(1):140. 28732508 10.1186/s12944-017-0536-0 PMC5521143

[73] Aoki J, Taira A, Takanezawa Y, Kishi Y, Hama K, Kishimoto T, et al. Serum lysophosphatidic acid is produced through diverse phospholipase pathways. J Biol Chem. 2002;277(50):48737–44. 12354767 10.1074/jbc.M206812200

[74] le Balle F, Simon MF, Meijer S, Fourcade O, Chap H. Membrane sidedness of biosynthetic pathways involved in the production of lysophosphatidic acid. Adv Enzyme Regul. 1999;39:275–84. 10470378 10.1016/s0065-2571(98)00024-7

[75] Sanchez-Marin L, Ladron de Guevara-Miranda D, Manas-Padilla MC, Alen F, Moreno-Fernandez RD, Diaz-Navarro C, et al. Systemic blockade of LPA1/3 lysophosphatidic acid receptors by ki16425 modulates the effects of ethanol on the brain and behavior. Neuropharmacology. 2018;133:189–201. 29378212 10.1016/j.neuropharm.2018.01.033

[76] McKlveen JM, Myers B, Herman JP. The medial prefrontal cortex: coordinator of autonomic, neuroendocrine and behavioural responses to stress. J Neuroendocrinol. 2015;27(6):446–56. 25737097 10.1111/jne.12272 PMC4580281

[77] Auclair N, Otani S, Soubrie P, Crepel F. Cannabinoids modulate synaptic strength and plasticity at glutamatergic synapses of rat prefrontal cortex pyramidal neurons. J Neurophysiol. 2000;83(6):3287–93. 10848548 10.1152/jn.2000.83.6.3287

[78] McLaughlin RJ, Hill MN, Gorzalka BB. A critical role for prefrontocortical endocannabinoid signaling in the regulation of stress and emotional behavior. Neurosci Biobehav Rev. 2014;42:116–31. 24582908 10.1016/j.neubiorev.2014.02.006

[79] Grabon W, Rheims S, Smith J, Bodennec J, Belmeguenai A, Bezin L. CB2 receptor in the CNS: from immune and neuronal modulation to behavior. Neurosci Biobehav Rev. 2023;150:105226. 37164044 10.1016/j.neubiorev.2023.105226

[80] Marco EM, Echeverry-Alzate V, Lopez-Moreno JA, Gine E, Penasco S, Viveros MP. Consequences of early life stress on the expression of endocannabinoid-related genes in the rat brain. Behav Pharmacol. 2014;25(5–6):547–56. 25083571 10.1097/FBP.0000000000000068

[81] Premachandran H, Zhao M, Arruda-Carvalho M. Sex differences in the development of the rodent corticolimbic system. Front Neurosci. 2020;14:583477. 33100964 10.3389/fnins.2020.583477 PMC7554619

[82] Leistner C, Menke A. Hypothalamic-pituitary-adrenal axis and stress. Handb Clin Neurol. 2020;175:55–64. 33008543 10.1016/B978-0-444-64123-6.00004-7

[83] Simone JJ, Green MR, McCormick CM. Endocannabinoid system contributions to sex-specific adolescent neurodevelopment. Prog Neuropsychopharmacol Biol Psychiatry. 2022;113:110438. 34534603 10.1016/j.pnpbp.2021.110438

[84] Rincon-Cortes M. Sex differences in addiction-relevant behavioral outcomes in rodents following early life stress. Addict Neurosci. 2023. 10.1016/j.addicn.2023.100067. 37101684 10.1016/j.addicn.2023.100067 PMC10124992

