# Supplementary material for: Maternal deprivation and adolescent alcohol exposure induce sex-dependent alterations in stress-related behavior and lipid signaling in rats
Source: Biol Sex Differ. 2026 Jun 7;17:117. doi: 10.1186/s13293-026-00937-2 (PMC13255284; doi:10.1186/s13293-026-00937-2)
Supplement: Supplementary file 1 — Supplementary Material 1 [file 13293_2026_937_MOESM1_ESM.pptx]

## Slide 1
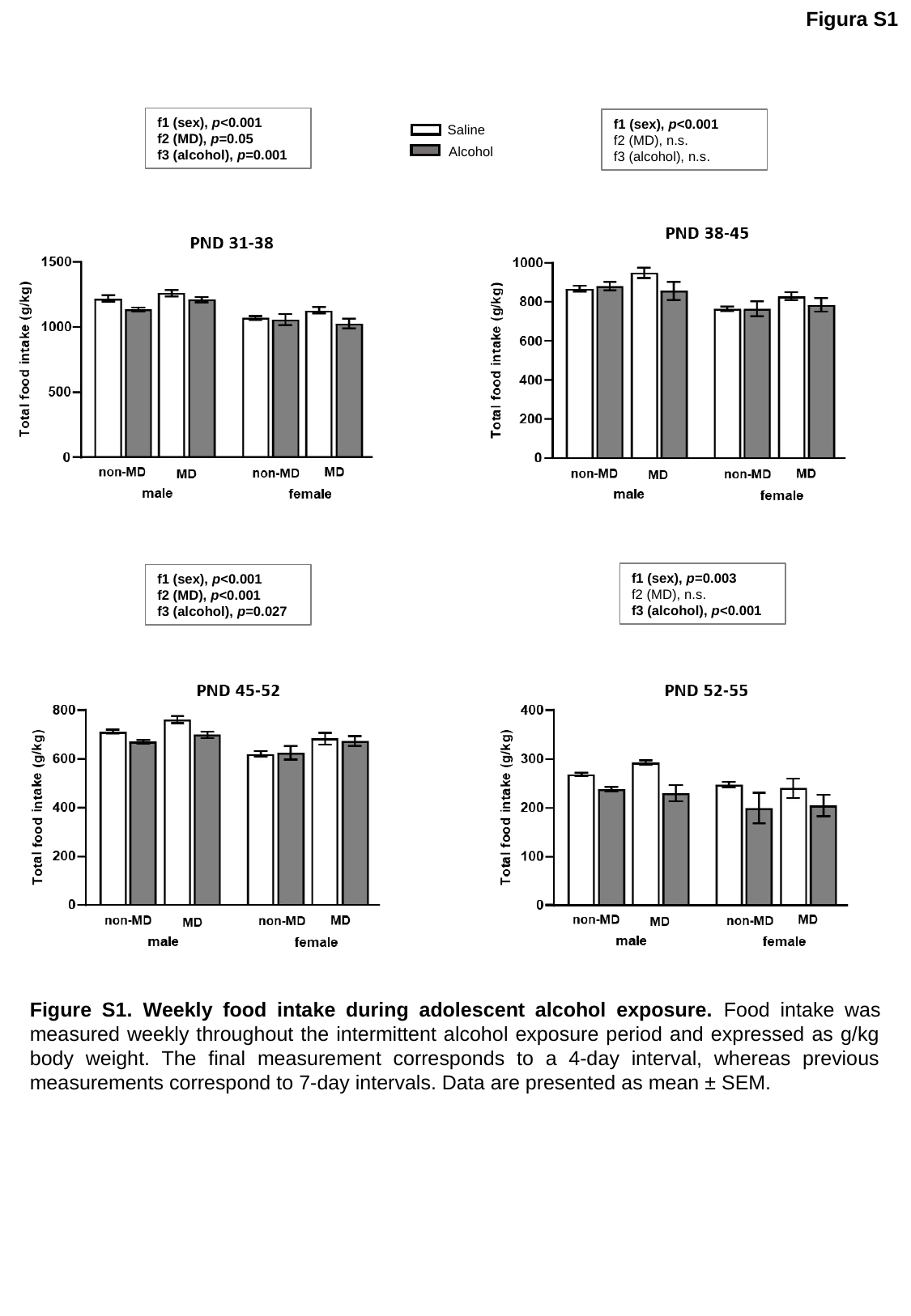

Figura S1
f1 (sex), p<0.001
f2 (MD), p=0.05
f3 (alcohol), p=0.001
f1 (sex), p<0.001
f2 (MD), n.s.
f3 (alcohol), n.s.
Saline
Alcohol
f1 (sex), p=0.003
f2 (MD), n.s.
f3 (alcohol), p<0.001
f1 (sex), p<0.001
f2 (MD), p<0.001
f3 (alcohol), p=0.027
Figure S1. Weekly food intake during adolescent alcohol exposure. Food intake was measured weekly throughout the intermittent alcohol exposure period and expressed as g/kg body weight. The final measurement corresponds to a 4-day interval, whereas previous measurements correspond to 7-day intervals. Data are presented as mean ± SEM.
