# Supplementary material for: Maternal deprivation and adolescent alcohol exposure induce sex-dependent alterations in stress-related behavior and lipid signaling in rats
Source: Biol Sex Differ. 2026 Jun 7;17:117. doi: 10.1186/s13293-026-00937-2 (PMC13255284; doi:10.1186/s13293-026-00937-2)
Supplement: Supplementary file 2 — Supplementary Material 2 [file 13293_2026_937_MOESM2_ESM.docx]

**Table S1.** Primer references for TaqMan® Gene Expression Assays (Applied Biosystems).

| Gene description | Assay ID | No accession  GenBank | Amplicon Length |
| --- | --- | --- | --- |
| *Actb* | Rn00667869_m1 | NM_031144.3 | 91 |
| *Cnr1* | Rn02758689_s1 | NM_012784.4 | 92 |
| *Cnr2* | Rn04342831_s1 | NM_020543.4 | 99 |
| *Daglα* | Rn01454304_m1 | NM_001005886.1 | 67 |
| *Daglβ* | Rn01453771_m1 | NM_001107120.1 | 98 |
| *Enpp2* | Rn01505088_m1 | NM_057104.2 | 66 |
| *Faah* | Rn00577086_m1 | NM_024132.3 | 63 |
| *Lpar1* | Rn00588435_m1 | NM_053936.3 | 67 |
| *Mgll* | Rn00593297_m1 | NM_138502.2 | 78 |
| *Napepld* | Rn01786262_m1 | NM_199381.1 | 71 |
| *Ppara* | Rn00566193_m1 | NM_013196.1 | 98 |
