# Supplementary material for: Maternal deprivation and adolescent alcohol exposure induce sex-dependent alterations in stress-related behavior and lipid signaling in rats
Source: Biol Sex Differ. 2026 Jun 7;17:117. doi: 10.1186/s13293-026-00937-2 (PMC13255284; doi:10.1186/s13293-026-00937-2)
Supplement: Supplementary file 3 — Supplementary Material 3 [file 13293_2026_937_MOESM3_ESM.docx]

**Table S2.** Complete ANOVA statistics for all experimental variables

| **Variable** | **Factor** | **F (DFn, DFd)** | ***p* value** | **ηp²** |
| --- | --- | --- | --- | --- |
| **BW gain after MD (fig. 1B)** | *f1 (sex)* | F (1, 64) = 6.250 | **0.0150** | 0.09 |
|  | *f2 (MD)* | F (1, 64) = 1919 | **< 0.001** | 0.97 |
|  | *f1 x f2* | F (1, 64) = 1.142 | 0.2893 | 0.02 |
| **BAC (fig. 1C)** | *f1 (sex)* | F (1, 61) = 36.14 | **< 0.001** | 0.37 |
|  | *f2 (MD)* | F (1, 61) = 2.069 | 0.1555 | 0.03 |
|  | *f3 (time)* | F (1, 61) = 12.32 | **< 0.001** | 0.17 |
|  | *f1 x f2* | F (1, 61) = 7.057 | **0.0101** | 0.10 |
|  | *f1 x f3* | F (1, 61) = 1.750 | 0.1909 | 0.03 |
|  | *f2 x f3* | F (1, 61) = 0.2245 | 0.6373 | 0.00 |
|  | *f1 x f2 x f3* | F (1, 61) = 0.4371 | 0.5110 | 0.01 |
| **BW gain after alcohol exposure (fig. 1D)** | *f1 (sex)* | F (1, 57) = 723.3 | **< 0.001** | 0.93 |
|  | *f2 (MD)* | F (1, 57) = 1.266 | 0.2653 | 0.02 |
|  | *f3 (alcohol)* | F (1, 57) = 0.0589 | 0.8096 | 0.00 |
|  | *f1 x f2* | F (1, 57) = 0.9410 | 0.3361 | 0.02 |
|  | *f1 x f3* | F (1, 57) = 0.7334 | 0.3954 | 0.01 |
|  | *f2 x f3* | F (1, 57) = 0.0930 | 0.7615 | 0.00 |
|  | *f1 x f2 x f3* | F (1, 57) = 3.479 | 0.0673 | 0.06 |
| **Total food intake (fig. 1E)** | *f1 (sex)* | F (1, 57) = 27.28 | **< 0.001** | 0.32 |
|  | *f2 (MD)* | F (1, 57) = 6.608 | **0.0128** | 0.10 |
|  | *f3 (alcohol)* | F (1, 57) = 13.79 | **< 0.001** | 0.19 |
|  | *f1 x f2* | F (1, 57) = 0.0841 | 0.7728 | 0.00 |
|  | *f1 x f3* | F (1, 57) = 0.2571 | 0.6141 | 0.00 |
|  | *f2 x f3* | F (1, 57) = 1.464 | 0.2313 | 0.03 |
|  | *f1 x f2 x f3* | F (1, 57) = 0.1180 | 0.7324 | 0.00 |
