# Supplementary material for: Maternal deprivation and adolescent alcohol exposure induce sex-dependent alterations in stress-related behavior and lipid signaling in rats
Source: Biol Sex Differ. 2026 Jun 7;17:117. doi: 10.1186/s13293-026-00937-2 (PMC13255284; doi:10.1186/s13293-026-00937-2)
Supplement: Supplementary file 4 — Supplementary Material 4 [file 13293_2026_937_MOESM4_ESM.docx]

**Table S3.** Complete ANOVA statistics for all experimental variables

| **Variable** | **Factor** | **F (DFn, DFd)** | ***p* value** | **ηp²** |
| --- | --- | --- | --- | --- |
| **Immobility time (fig. 2A)** | *f1 (sex)* | F (1, 57) = 3.548 | 0.0647 | 0.06 |
|  | *f2 (MD)* | F (1, 57) = 2.359 | 0.1301 | 0.04 |
|  | *f3 (alcohol)* | F (1, 57) = 0.1304 | 0.7194 | 0.00 |
|  | *f1 x f2* | F (1, 57) = 3.124 | 0.0825 | 0.05 |
|  | *f1 x f3* | F (1, 57) = 0.0021 | 0.9638 | 0.00 |
|  | *f2 x f3* | F (1, 57) = 2.052 | 0.1574 | 0.03 |
|  | *f1 x f2 x f3* | F (1, 57) = 3.477 | 0.0674 | 0.06 |
| **Escape behaviors (fig. 2B)** | *f1 (sex)* | F (1, 57) = 7.071 | **0.0102** | 0.11 |
|  | *f2 (MD)* | F (1, 57) = 0.1012 | 0.7515 | 0.00 |
|  | *f3 (alcohol)* | F (1, 57) = 4.488 | **0.0385** | 0.07 |
|  | *f1 x f2* | F (1, 57) = 13.53 | **< 0.001** | 0.19 |
|  | *f1 x f3* | F (1, 57) = 0.4373 | 0.5111 | 0.01 |
|  | *f2 x f3* | F (1, 57) = 8.086 | **0.006** | 0.12 |
|  | *f1 x f2 x f3* | F (1, 57) = 1.514 | 0.2235 | 0.03 |
| **OTR (fig. 2C)** | *f1 (sex)* | F (1, 57) = 8.740 | **0.0045** | 0.13 |
|  | *f2 (MD)* | F (1, 57) = 6.106 | **0.0165** | 0.10 |
|  | *f3 (alcohol)* | F (1, 57) = 0.1912 | 0.6635 | 0.00 |
|  | *f1 x f2* | F (1, 57) = 0.1341 | 0.7156 | 0.00 |
|  | *f1 x f3* | F (1, 57) = 0.1963 | 0.6594 | 0.00 |
|  | *f2 x f3* | F (1, 57) = 1.734 | 0.1932 | 0.03 |
|  | *f1 x f2 x f3* | F (1, 57) = 0.0002 | 0.9868 | 0.00 |
| **Distance open arms (fig. 2D)** | *f1 (sex)* | F (1, 57) = 8.791 | **0.0044** | 0.13 |
|  | *f2 (MD)* | F (1, 57) = 0.0028 | 0.9580 | 0.00 |
|  | *f3 (alcohol)* | F (1, 57) = 1.375 | 0.2458 | 0.02 |
|  | *f1 x f2* | F (1, 57) = 1.432 | 0.2364 | 0.02 |
|  | *f1 x f3* | F (1, 57) = 0.6467 | 0.4246 | 0.01 |
|  | *f2 x f3* | F (1, 57) = 1.882 | 0.1755 | 0.03 |
|  | *f1 x f2 x f3* | F (1, 57) = 0.1823 | 0.6711 | 0.00 |
| **Open arm entries (fig. 2E)** | *f1 (sex)* | F (1, 57) = 27.43 | **< 0.001** | 0.32 |
|  | *f2 (MD)* | F (1, 57) = 1.372 | 0.2463 | 0.02 |
|  | *f3 (alcohol)* | F (1, 57) = 0.7598 | 0.3871 | 0.01 |
|  | *f1 x f2* | F (1, 57) = 0.3622 | 0.5497 | 0.01 |
|  | *f1 x f3* | F (1, 57) = 0.0316 | 0.8595 | 0.00 |
|  | *f2 x f3* | F (1, 57) = 2.290 | 0.1357 | 0.04 |
|  | *f1 x f2 x f3* | F (1, 57) = 1.072 | 0.3049 | 0.02 |
| **Time in closed arms (fig. 2F)** | *f1 (sex)* | F (1, 57) = 5.092 | **0.0279** | 0.08 |
|  | *f2 (MD)* | F (1, 57) = 1.973 | 0.1655 | 0.03 |
|  | *f3 (alcohol)* | F (1, 57) = 1.327 | 0.2542 | 0.02 |
|  | *f1 x f2* | F (1, 57) = 0.00107 | 0.9740 | 0.00 |
|  | *f1 x f3* | F (1, 57) = 0.07451 | 0.7859 | 0.00 |
|  | *f2 x f3* | F (1, 57) = 0.7369 | 0.3943 | 0.01 |
|  | *f1 x f2 x f3* | F (1, 57) = 0.00029 | 0.9865 | 0.00 |
| **Total distance (fig. 2G)** | *f1 (sex)* | F (1, 57) = 3.562 | 0.6653 | 0.06 |
|  | *f2 (MD)* | F (1, 57) = 0.1891 | 0.0554 | 0.00 |
|  | *f3 (alcohol)* | F (1, 57) = 3.824 | 0.4478 | 0.06 |
|  | *f1 x f2* | F (1, 57) = 0.5843 | 0.8503 | 0.01 |
|  | *f1 x f3* | F (1, 57) = 0.03594 | 0.6176 | 0.00 |
|  | *f2 x f3* | F (1, 57) = 0.2520 | 0.6505 | 0.00 |
|  | *f1 x f2 x f3* | F (1, 57) = 0.2074 | 0.6653 | 0.00 |
| **Time in center (fig. 2H)** | *f1 (sex)* | F (1, 57) = 0.09961 | 0.7534 | 0.00 |
|  | *f2 (MD)* | F (1, 57) = 0.7778 | 0.3815 | 0.01 |
|  | *f3 (alcohol)* | F (1, 57) = 0.1321 | 0.7176 | 0.00 |
|  | *f1 x f2* | F (1, 57) = 0.5920 | 0.4448 | 0.01 |
|  | *f1 x f3* | F (1, 57) = 0.3720 | 0.5444 | 0.01 |
|  | *f2 x f3* | F (1, 57) = 1.423 | 0.2379 | 0.02 |
|  | *f1 x f2 x f3* | F (1, 57) = 0.01046 | 0.9189 | 0.00 |
