# Supplementary material for: Maternal deprivation and adolescent alcohol exposure induce sex-dependent alterations in stress-related behavior and lipid signaling in rats
Source: Biol Sex Differ. 2026 Jun 7;17:117. doi: 10.1186/s13293-026-00937-2 (PMC13255284; doi:10.1186/s13293-026-00937-2)
Supplement: Supplementary file 5 — Supplementary Material 5 [file 13293_2026_937_MOESM5_ESM.docx]

**Table S4.** Complete ANOVA statistics for all experimental variables

| **Variable** | **Factor** | **F (DFn, DFd)** | ***p* value** | **ηp²** |
| --- | --- | --- | --- | --- |
| **Corticosterone (fig. 3A)** | *f1 (sex)* | F (1, 56) = 3.781 | 0.0569 | 0.06 |
|  | *f2 (MD)* | F (1, 56) = 4.024 | 0.0517 | 0.07 |
|  | *f3 (alcohol)* | F (1, 56) = 5.147 | **0.0272** | 0.08 |
|  | *f1 x f2* | F (1, 56) = 0.5264 | 0.4712 | 0.01 |
|  | *f1 x f3* | F (1, 56) = 1.645 | 0.2049 | 0.03 |
|  | *f2 x f3* | F (1, 56) = 0.1323 | 0.7174 | 0.00 |
|  | *f1 x f2 x f3* | F (1, 56) = 0.1237 | 0.7264 | 0.00 |
| **2-LG (fig. 3B)** | *f1 (sex)* | F (1, 57) = 44.90 | **< 0.001** | 0.44 |
|  | *f2 (MD)* | F (1, 57) = 2.871 | 0.0956 | 0.05 |
|  | *f3 (alcohol)* | F (1, 57) = 10.95 | **0.0016** | 0.16 |
|  | *f1 x f2* | F (1, 57) = 10.94 | **0.0016** | 0.16 |
|  | *f1 x f3* | F (1, 57) = 10.10 | **0.0024** | 0.15 |
|  | *f2 x f3* | F (1, 57) = 6.233 | **0.0155** | 0.10 |
|  | *f1 x f2 x f3* | F (1, 57) = 4.933 | **0.0303** | 0.08 |
| **LEA (fig. 3C)** | *f1 (sex)* | F (1, 57) = 4.451 | **0.0393** | 0.07 |
|  | *f2 (MD)* | F (1, 57) = 1.547 | 0.2186 | 0.03 |
|  | *f3 (alcohol)* | F (1, 57) = 0.9219 | 0.3410 | 0.02 |
|  | *f1 x f2* | F (1, 57) = 4.296 | **0.0427** | 0.07 |
|  | *f1 x f3* | F (1, 57) = 0.0587 | 0.8094 | 0.00 |
|  | *f2 x f3* | F (1, 57) = 6.260 | **0.0152** | 0.10 |
|  | *f1 x f2 x f3* | F (1, 57) = 0.6912 | 0.4092 | 0.01 |
| **2-OG (fig. 3D)** | *f1 (sex)* | F (1, 57) = 51.24 | **< 0.001** | 0.47 |
|  | *f2 (MD)* | F (1, 57) = 1.410 | 0.2399 | 0.02 |
|  | *f3 (alcohol)* | F (1, 57) = 6.107 | **0.0165** | 0.10 |
|  | *f1 x f2* | F (1, 57) = 7.576 | **0.0079** | 0.12 |
|  | *f1 x f3* | F (1, 57) = 3.281 | 0.0753 | 0.05 |
|  | *f2 x f3* | F (1, 57) = 1.674 | 0.2009 | 0.03 |
|  | *f1 x f2 x f3* | F (1, 57) = 1.928 | 0.1704 | 0.03 |
| **OEA (fig. 3E)** | *f1 (sex)* | F (1, 57) = 19.27 | **< 0.001** | 0.25 |
|  | *f2 (MD)* | F (1, 57) = 0.1120 | 0.7391 | 0.00 |
|  | *f3 (alcohol)* | F (1, 57) = 1.084 | 0.3023 | 0.02 |
|  | *f1 x f2* | F (1, 57) = 5.128 | **0.0274** | 0.08 |
|  | *f1 x f3* | F (1, 57) = 0.4033 | 0.5280 | 0.01 |
|  | *f2 x f3* | F (1, 57) = 1.419 | 0.2386 | 0.02 |
|  | *f1 x f2 x f3* | F (1, 57) = 0.0193 | 0.8899 | 0.00 |
| **2-AG (fig. 3F)** | *f1 (sex)* | F (1, 57) = 1.909 | 0.1724 | 0.03 |
|  | *f2 (MD)* | F (1, 57) = 11.94 | **< 0.001** | 0.17 |
|  | *f3 (alcohol)* | F (1, 57) = 1.770 | 0.1887 | 0.03 |
|  | *f1 x f2* | F (1, 57) = 4.464 | **0.0390** | 0.07 |
|  | *f1 x f3* | F (1, 57) = 0.5136 | 0.4765 | 0.01 |
|  | *f2 x f3* | F (1, 57) = 5.549 | **0.0220** | 0.09 |
|  | *f1 x f2 x f3* | F (1, 57) = 4.141 | **0.0465** | 0.07 |
| **AEA (fig. 3G)** | *f1 (sex)* | F (1, 57) = 4.430 | **0.0397** | 0.07 |
|  | *f2 (MD)* | F (1, 57) = 9.683e-005 | 0.9922 | 0.00 |
|  | *f3 (alcohol)* | F (1, 57) = 0.000167 | 0.9898 | 0.00 |
|  | *f1 x f2* | F (1, 57) = 1.562 | 0.2164 | 0.03 |
|  | *f1 x f3* | F (1, 57) = 0.0198 | 0.8886 | 0.00 |
|  | *f2 x f3* | F (1, 57) = 0.8263 | 0.3672 | 0.01 |
|  | *f1 x f2 x f3* | F (1, 57) = 1.111 | 0.2964 | 0.02 |
| **LPA (fig. 3H)** | *f1 (sex)* | F (1, 57) = 10.96 | **0.0016** | 0.16 |
|  | *f2 (MD)* | F (1, 57) = 11.15 | **0.0015** | 0.16 |
|  | *f3 (alcohol)* | F (1, 57) = 2.899 | 0.0941 | 0.05 |
|  | *f1 x f2* | F (1, 57) = 2.070 | 0.1557 | 0.04 |
|  | *f1 x f3* | F (1, 57) = 3.456 | 0.0682 | 0.06 |
|  | *f2 x f3* | F (1, 57) = 0.8536 | 0.3594 | 0.01 |
|  | *f1 x f2 x f3* | F (1, 57) = 1.358 | 0.2487 | 0.02 |
| **Autotaxin (fig. 3I)** | *f1 (sex)* | F (1, 57) = 3.499 | 0.0665 | 0.06 |
|  | *f2 (MD)* | F (1, 57) = 0.3458 | 0.5588 | 0.01 |
|  | *f3 (alcohol)* | F (1, 57) = 4.650 | **0.0353** | 0.08 |
|  | *f1 x f2* | F (1, 57) = 0.0567 | 0.8127 | 0.00 |
|  | *f1 x f3* | F (1, 57) = 0.0833 | 0.7740 | 0.00 |
|  | *f2 x f3* | F (1, 57) = 9.568 | **0.0031** | 0.14 |
|  | *f1 x f2 x f3* | F (1, 57) = 0.0894 | 0.7661 | 0.00 |
