# Supplementary material for: Maternal deprivation and adolescent alcohol exposure induce sex-dependent alterations in stress-related behavior and lipid signaling in rats
Source: Biol Sex Differ. 2026 Jun 7;17:117. doi: 10.1186/s13293-026-00937-2 (PMC13255284; doi:10.1186/s13293-026-00937-2)
Supplement: Supplementary file 6 — Supplementary Material 6 [file 13293_2026_937_MOESM6_ESM.docx]

**Table S5.** Complete ANOVA statistics for all experimental variables

| **Variable** | **Factor** | **F (DFn, DFd)** | ***p* value** | **ηp²** |
| --- | --- | --- | --- | --- |
| ***Cnr1* mRNA (fig. 4A)** | *f1 (sex)* | F (1, 40) = 21.07 | **< 0.001** | 0.35 |
|  | *f2 (MD)* | F (1, 40) = 795.2 | **< 0.001** | 0.95 |
|  | *f3 (alcohol)* | F (1, 40) = 147.2 | **< 0.001** | 0.79 |
|  | *f1 x f2* | F (1, 40) = 17.63 | **< 0.001** | 0.31 |
|  | *f1 x f3* | F (1, 40) = 0.8187 | 0.3710 | 0.02 |
|  | *f2 x f3* | F (1, 40) = 25.84 | **< 0.001** | 0.39 |
|  | *f1 x f2 x f3* | F (1, 40) = 0.2426 | 0.6250 | 0.01 |
| ***Cnr2* mRNA (fig. 4B)** | *f1 (sex)* | F (1, 40) = 3.413 | 0.0721 | 0.08 |
|  | *f2 (MD)* | F (1, 40) = 6.207 | **0.0170** | 0.13 |
|  | *f3 (alcohol)* | F (1, 40) = 0.0322 | 0.8585 | 0.00 |
|  | *f1 x f2* | F (1, 40) = 0.9433 | 0.3373 | 0.02 |
|  | *f1 x f3* | F (1, 40) = 0.6103 | 0.4393 | 0.02 |
|  | *f2 x f3* | F (1, 40) = 0.0001 | 0.9916 | 0.00 |
|  | *f1 x f2 x f3* | F (1, 40) = 0.4565 | 0.5032 | 0.01 |
| ***Ppara* mRNA (fig. 4C)** | *f1 (sex)* | F (1, 40) = 0,2729 | 0.6043 | 0.01 |
|  | *f2 (MD)* | F (1, 40) = 2.070 | 0.1580 | 0.05 |
|  | *f3 (alcohol)* | F (1, 40) = 6.352 | **0.0158** | 0.14 |
|  | *f1 x f2* | F (1, 40) = 0.1855 | 0.6690 | 0.00 |
|  | *f1 x f3* | F (1, 40) = 0.0021 | 0.9637 | 0.00 |
|  | *f2 x f3* | F (1, 40) = 4.798 | **0.0344** | 0.11 |
|  | *f1 x f2 x f3* | F (1, 40) = 0.7903 | 0.3793 | 0.02 |
| ***Lpar1* mRNA (fig. 4D)** | *f1 (sex)* | F (1, 40) = 1.006 | 0.3218 | 0.02 |
|  | *f2 (MD)* | F (1, 40) = 0.4104 | 0.5254 | 0.01 |
|  | *f3 (alcohol)* | F (1, 40) = 1.642 | 0.2075 | 0.04 |
|  | *f1 x f2* | F (1, 40) = 0.7785 | 0.3829 | 0.02 |
|  | *f1 x f3* | F (1, 40) = 5.386 | **0.0255** | 0.12 |
|  | *f2 x f3* | F (1, 40) = 1.833 | 0.1834 | 0.04 |
|  | *f1 x f2 x f3* | F (1, 40) = 3.555 | 0.0666 | 0.08 |
