# Supplementary material for: Maternal deprivation and adolescent alcohol exposure induce sex-dependent alterations in stress-related behavior and lipid signaling in rats
Source: Biol Sex Differ. 2026 Jun 7;17:117. doi: 10.1186/s13293-026-00937-2 (PMC13255284; doi:10.1186/s13293-026-00937-2)
Supplement: Supplementary file 7 — Supplementary Material 7 [file 13293_2026_937_MOESM7_ESM.docx]

**Table S6.** Complete ANOVA statistics for all experimental variables

| **Variable** | **Factor** | **F (DFn, DFd)** | ***p* value** | ***ηp²*** |
| --- | --- | --- | --- | --- |
| ***Dagla* mRNA (fig. 5A)** | *f1 (sex)* | F (1, 40) = 0.5554 | 0.4605 | 0.01 |
|  | *f2 (MD)* | F (1, 40) = 167.0 | **< 0.001** | 0.81 |
|  | *f3 (alcohol)* | F (1, 40) = 9.857 | **0.0032** | 0.20 |
|  | *f1 x f2* | F (1, 40) = 7.623 | **0.0087** | 0.16 |
|  | *f1 x f3* | F (1, 40) = 2.298 | 0.1374 | 0.05 |
|  | *f2 x f3* | F (1, 40) = 7.032 | **0.0114** | 0.15 |
|  | *f1 x f2 x f3* | F (1, 40) = 3.262 | 0.0785 | 0.08 |
| ***Daglb* mRNA (fig. 5B)** | *f1 (sex)* | F (1, 40) = 1.972 | 0.1680 | 0.05 |
|  | *f2 (MD)* | F (1, 40) = 36.97 | **< 0.001** | 0.48 |
|  | *f3 (alcohol)* | F (1, 40) = 9.365 | **0.0039** | 0.19 |
|  | *f1 x f2* | F (1, 40) = 0.00987 | 0.9214 | 0.00 |
|  | *f1 x f3* | F (1, 40) = 0.2468 | 0.6221 | 0.01 |
|  | *f2 x f3* | F (1, 40) = 0.1141 | 0.7373 | 0.00 |
|  | *f1 x f2 x f3* | F (1, 40) = 0.0774 | 0.7823 | 0.00 |
| ***Napepld* mRNA (fig. 5C)** | *f1 (sex)* | F (1, 40) = 0.5565 | 0.4600 | 0.01 |
|  | *f2 (MD)* | F (1, 40) = 1.276 | 0.2653 | 0.03 |
|  | *f3 (alcohol)* | F (1, 40) = 1.047 | 0.3124 | 0.03 |
|  | *f1 x f2* | F (1, 40) = 0.0955 | 0.7589 | 0.00 |
|  | *f1 x f3* | F (1, 40) = 1.450 | 0.2356 | 0.03 |
|  | *f2 x f3* | F (1, 40) = 1.608 | 0.2120 | 0.04 |
|  | *f1 x f2 x f3* | F (1, 40) = 0.1817 | 0.6722 | 0.00 |
| ***Enpp2* mRNA (fig. 5D)** | *f1 (sex)* | F (1, 40) = 0.3002 | 0.5868 | 0.01 |
|  | *f2 (MD)* | F (1, 40) = 8.118 | **0.0069** | 0.17 |
|  | *f3 (alcohol)* | F (1, 40) = 3.129 | 0.0845 | 0.07 |
|  | *f1 x f2* | F (1, 40) = 0.6627 | 0.4204 | 0.02 |
|  | *f1 x f3* | F (1, 40) = 0.8531 | 0.3612 | 0.02 |
|  | *f2 x f3* | F (1, 40) = 1.004 | 0.3224 | 0.02 |
|  | *f1 x f2 x f3* | F (1, 40) = 2.550 | 0.1182 | 0.06 |
| ***Mgll* mRNA (fig. 5E)** | *f1 (sex)* | F (1, 40) = 4.187 | **0.0474** | 0.09 |
|  | *f2 (MD)* | F (1, 40) = 23.91 | **< 0.001** | 0.37 |
|  | *f3 (alcohol)* | F (1, 40) = 4.602 | **0.0381** | 0.10 |
|  | *f1 x f2* | F (1, 40) = 0.2916 | 0.5922 | 0.01 |
|  | *f1 x f3* | F (1, 40) = 0.06895 | 0.7942 | 0.00 |
|  | *f2 x f3* | F (1, 40) = 0.05422 | 0.8171 | 0.00 |
|  | *f1 x f2 x f3* | F (1, 40) = 0.0454 | 0.8324 | 0.00 |
| ***Faah* mRNA (fig. 5F)** | *f1 (sex)* | F (1, 40) = 0.7933 | 0.3784 | 0.02 |
|  | *f2 (MD)* | F (1, 40) = 3.149 | 0.0836 | 0.07 |
|  | *f3 (alcohol)* | F (1, 40) = 0.0138 | 0.9071 | 0.00 |
|  | *f1 x f2* | F (1, 40) = 5.350 | **0.0259** | 0.12 |
|  | *f1 x f3* | F (1, 40) = 2.204 | 0.1455 | 0.05 |
|  | *f2 x f3* | F (1, 40) = 9.783 | **0.0033** | 0.20 |
|  | *f1 x f2 x f3* | F (1, 40) = 0.7212 | 0.4008 | 0.02 |
